# Prevalence and clinical characterization of malnutrition, sarcopenia and sarcopenic obesity in older adults with cardiometabolic diseases: SENIOR cross-sectional study

**DOI:** 10.3389/fnut.2026.1927628

**Published:** 2026-10-28

**Authors:** Sara Santero, Federica Bolpagni, Sara Boussetta, Francesca Coperchini, Ginevra Biino, Nagaia Madini, Cecilia Ricciardi Rizzo, Flavia Magri, Mario Rotondi, Alessandra Vincenti, Alice Beretta, Giulia Viroli, Matteo Manuelli, Maria Luisa Fonte, Ilaria Di Napoli, Donatella Corvino, Pasquale De Cata, Antonio Di Sabatino, Anna Giulia Falchi, Ilaria Ferrari, Alessandra Marchese, Marco Vincenzo Lenti, Federica Lepore, Francesco Mordà, Mariangela Delliponti, Maria Emilia Paladino, Michele A Riva, Hellas Cena

**Affiliations:** 1 Laboratory of Dietetics and Clinical Nutrition, Department of Public Health, Experimental and Forensic Medicine, University of Pavia, Pavia, Italy; 2 Istituti Clinici Scientifici Maugeri IRCCS, Unit of Clinical Nutrition, Pavia, Italy; 3 National Biodiversity Future Center (NBFC), Palermo, Italy; 4 Department of Earth and Marine Sciences (DiSTeM), University of Palermo, Palermo, Italy; 5 Unit of Endocrinology and Metabolism, Laboratory for Endocrine Disruptors, Istituti Clinici Scientifici Maugeri IRCCS, Pavia, Italy; 6 Department of Internal Medicine and Therapeutics, University of Pavia, Pavia, Italy; 7 Institute of Molecular Genetics, National Research Council of Italy, Pavia, Italy; 8 Quironsalud Hospital Sagrado Corazón, Sevilla, Spain; 9 Istituti Clinici Scientifici Maugeri IRCCS, Unit of Internal Medicine, Pavia, Italy; 10 First Department of Internal Medicine, Fondazione IRCCS Policlinico San Matteo, Pavia, Italy; 11 SC Coordinamento, Supporto e Monitoraggio della Ricerca clinica e Sperimentale, Fondazione IRCCS Policlinico San Matteo, Pavia, Italy; 12 School of Medicine and Surgery, University of Milano-Bicocca, Monza, Italy; 13 Department of Occupational Health, Fondazione IRCCS San Gerardo dei Tintori, Monza, Italy

**Keywords:** cardiometabolic diseases, cardiovascular diseases, inflammation, malnutrition, older adults, sarcopenia, sarcopenic obesity

## Abstract

Malnutrition, sarcopenia, and sarcopenic obesity may coexist with excess adiposity and cardiometabolic diseases in aging populations, but their combined burden and inflammatory profile remain insufficiently characterized. This study examined their prevalence and overlap in older adults with cardiometabolic diseases and explored associations with metabolic syndrome, multidimensional health-related factors and inflammation. In this cross-sectional study 303 Caucasian adults aged ≥ 65 years were recruited from a General Medicine ward and an Endocrinology and Metabolic Diseases outpatient clinic in Northern Italy. Malnutrition, sarcopenia, sarcopenic obesity, and metabolic syndrome were identified according to the GLIM, EWGSOP2, ESPEN/EASO, and IDF criteria, respectively. Sociodemographic, clinical, anthropometric, body composition, lifestyle, functional, psychological, and biochemical variables were collected using standardized procedures. CRP, WBC, platelet counts, prealbumin, uric acid, and circulating cytokines (IL-6, IL-10, TNF-α, and TGF-β) provided complementary information on inflammatory, nutritional, and metabolic status. The prevalence of malnutrition, sarcopenia, sarcopenic obesity, probable sarcopenia, and metabolic syndrome was 39.9%, 30.9%, 9.4%, 42.6%, and 60.1%, respectively, and was generally higher in inpatients. 13.2% of malnourished patients suffered from overweight or obesity, while malnutrition and sarcopenia overlapped in 29.2%. Older age, functional dependence, poorer physical health-related quality of life, and BMI class were associated with malnutrition or sarcopenia, whereas sarcopenic obesity was associated with age 75–84 years and low physical activity. Metabolic syndrome was significantly associated with malnutrition, sarcopenia, and sarcopenic obesity. Higher circulating IL-6 concentrations were observed in older participants and in those with sarcopenic obesity or metabolic syndrome. Malnutrition, sarcopenia, sarcopenic obesity, and metabolic syndrome are highly prevalent and overlapping conditions among older adults with cardiometabolic diseases, especially in hospitalized patients. Excess body weight does not exclude nutritional or muscle impairment, thus integrated assessment is needed to identify vulnerable older adults and guide targeted interventions.

## 1 Introduction

Population aging represents a major demographic shift, being the proportion of people aged ≥65 years projected to rise to nearly 12% by 2030 (1), particularly in Italy (2). This demographic transition is accompanied by an increasing burden of “geriatric syndromes,” including malnutrition, sarcopenia and sarcopenic obesity (3–5), which frequently co-exist and favor adverse clinical outcomes (6, 7).

The relevance of these issues is particularly evident in Italy, where malnutrition and sarcopenia are highly prevalent among older adults, especially in hospital settings. Studies applying GLIM criteria have reported malnutrition rates of approximately 37–46% among hospitalized older adults (8, 9), while sarcopenia prevalence has been estimated between 16–34% (10–12). In contrast, lower prevalence rates (3–14%) have been observed among community-dwelling older adults (13, 14). Moreover, sarcopenic obesity is emerging as a significant public health concern, potentially affecting more than 2 million individuals nationwide (15).

One of the main biological mechanisms linking aging to these geriatric syndromes is chronic low-grade inflammation and immunosenescence, commonly referred to as “inflammaging” (16). In this context, the balance between pro- and anti-inflammatory cytokines plays a pivotal role in driving healthy or unhealthy aging trajectories. Among these cytokines, interleukin-6 (IL-6) and Tumor Necrosis Factor-α (TNF-α) are the most extensively studied, in view of their involvement in aging and inflammatory processes but also in regulation of appetite, muscle homeostasis, nutrient sensing, and taste perception (16–18).

The clinical relevance of these geriatric syndromes extends beyond functional decline and inflammation, as they are also closely linked to cardiometabolic diseases. A high prevalence of cardiovascular disease and diabetes has been observed among malnourished hospitalized older adults (19), as well as poorer clinical outcomes (20, 21). Likewise, sarcopenia seems to be associated with cardiometabolic risk and poorer clinical outcomes in older adults with cardiovascular disease (22, 23). Furthermore, the bidirectional association between sarcopenia and diabetes has led to the recognition of sarcopenic diabetes as a distinct clinical entity (24). Although evidence on sarcopenic obesity remains heterogeneous, this condition may confer greater cardiometabolic risk, highlighting the need for refined obesity phenotyping in geriatric populations (22, 25).

This evidence of association between geriatric syndromes and cardiometabolic diseases is not unexpected since they share common pathophysiological pathways including inflammation, insulin resistance and altered body composition (Figure 1, see Supplementary materials for the complete list of references underlying the Figure). Their interaction may further amplify the risk of metabolic syndrome and other cardiometabolic complications, emphasizing the role of a comprehensive nutritional assessment in older adults.

**Figure 1:**
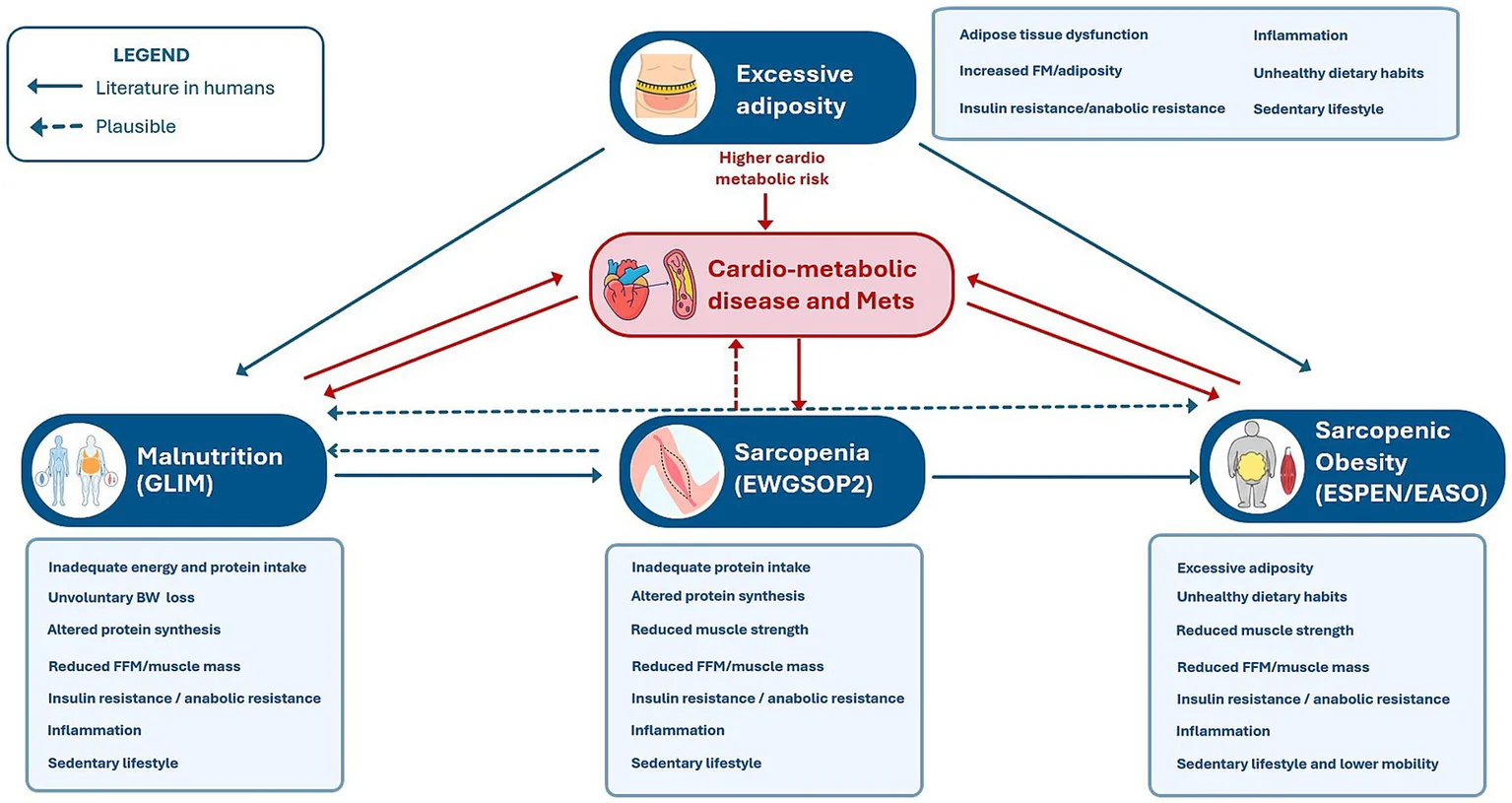
Interrelationships between malnutrition, sarcopenia, sarcopenic obesity, cardiometabolic diseases and metabolic syndrome. The figure summarizes the overlap among altered nutritional status, reduced fat-free mass and muscle strength, increased adiposity, central obesity and cardiometabolic risk. Nutritional and lifestyle-related factors are shown as potentially modifiable contributors, whereas inflammation, insulin resistance and anabolic resistance are depicted as shared pathogenic mechanisms across the conditions. The literature supporting this figure is provided in the Supplementary materials. ESPEN/EASO, European Society for Clinical Nutrition and Metabolism/European Association for the Study of Obesity; EWGSOP2, European Working Group on Sarcopenia in Older People 2; GLIM, Global Leadership Initiative on Malnutrition; Mets, metabolic syndrome.

Given the central role of inflammation in these interconnected conditions, dietary strategies with anti-inflammatory potential have attracted increasing interest in promoting healthy aging and reducing the risk of cardiometabolic and neurodegenerative diseases. The Mediterranean diet, characterized by high intake of plant-based foods, extra-virgin olive oil, and fish, exerts anti-inflammatory effects through modulation of inflammatory pathways, and beneficial effects on gut microbiota and metabolic function (26). Similarly, the Mediterranean-DASH Intervention for Neurodegenerative Delay (MIND) diet has been associated with improved cardiometabolic outcomes, although current evidence remains less robust than that supporting the Mediterranean diet (27).

Despite the growing evidence supporting the role of inflammation in aging populations (16), no Italian research has comprehensively evaluated the interplay between inflammation, cardiometabolic diseases, and metabolic syndrome in people affected by malnutrition, sarcopenia, and sarcopenic obesity. This represents an important gap, underscoring the need for integrated approaches to improve patient clinical phenotyping. On this basis, the aim of the present study is to assess the prevalence of malnutrition, sarcopenia, and sarcopenic obesity in older adults. Secondary objectives were to investigate their associations with lifestyle, functional status, inflammation and cardiometabolic comorbidities. To characterize the inflammatory and metabolic milieu of aging, we selected a complementary panel of biomarkers reflecting different but interconnected biological domains. CRP and white blood cell count were included as routinely available markers of systemic inflammation (28), whereas platelet count was considered as an additional marker of the thrombo-inflammatory milieu. Indeed, aging is associated with changes in platelet count and function, and growing evidence indicates that platelets participate not only in hemostasis and thrombosis but also in inflammatory and immune responses and age-related oxidative processes (29, 30). Prealbumin was assessed because of its relationship with nutritional status and its behavior as a negative acute-phase reactant, thus providing information at the interface between nutrition and inflammation (31, 32), whereas uric acid was included as a marker related to metabolic dysregulation and cardiometabolic risk (33). Finally, circulating cytokines were evaluated to provide a more specific characterization of the inflammatory network underlying inflammaging, including both pro-inflammatory mediators (IL-6 and TNF-α) and anti-inflammatory/immunoregulatory mediators (IL-10 and TGF-β) (16). Overall, this complementary panel was selected to capture different components of the inflammatory, nutritional, and metabolic alterations potentially relevant to malnutrition, sarcopenia, sarcopenic obesity, and cardiometabolic disease in older adults.

## 2 Materials and methods

### 2.1 Study design

The SENIOR (Sustainable Eating patterN to lImit malnutritiOn in oldeR adults) study was a multicenter, cross-sectional study involving three hospitals in Northern Italy: ICS Maugeri IRCCS (Pavia), IRCCS Policlinico San Matteo Foundation (Pavia), and IRCCS San Gerardo dei Tintori Foundation (Monza). The study protocol was registered on ClinicalTrials.gov (NCT05860556), providing a detailed description of the study design and collected variables. Also, it followed the STROBE reporting guidelines, including the relevant extensions STROBE-nut and STROBE-ME (34, 35).

The primary outcome of the study was the prevalence of malnutrition, defined according to the GLIM (Global Leadership Initiative on Malnutrition) criteria (3). Secondary outcomes included the prevalence of sarcopenia according to the EWGSOP2 consensus (4), sarcopenic obesity according to ESPEN/EASO (5), and malnutrition according to WHO classifications (overweight/obesity and underweight) (36). Furthermore, factors associated with malnutrition (GLIM) and sarcopenia (EWGSOP2) were planned to be examined across socio-demographic, lifestyle, health-related domains, and inflammatory, nutritional, and metabolic biomarkers.

### 2.2 Recruitment and study participants

This article focuses on results from inpatients admitted to the General Medicine ward and outpatients attending the Endocrinology and Metabolic Diseases clinic at ICS Maugeri IRCCS hospital in Pavia. Participants were consecutively recruited using a non-probability sampling approach from November 2023 to December 2025. A 3:1 ratio of inpatients to outpatients was maintained.

Eligibility criteria included: (i) older adults (age ≥65 years); (ii) admission to or attendance at one of the participating clinical units; and (iii) ability to understand and provide written informed consent, including consent for blood sample collection and laboratory analyses. Exclusion criteria were: (i) dysphagia; (ii) terminal illness; (iii) history of bariatric surgery; (iv) diagnosis of eating disorders (37); (v) clinically significant hepatic impairment (Child–Pugh class B or higher) (38); (vi) severe renal failure (estimated glomerular filtration rate eGFR <15 mL/min/1.73 m^2^) (39); (vii) active cancer not in remission and currently under oncological treatment; (viii) severe acute or chronic respiratory failure (partial pressure of oxygen pO2 < 60 mmHg or partial pressure of carbon dioxide pCO2 > 45 mmHg) (40); and (ix) severe cognitive impairment, defined as a Mini-Mental State Examination (MMSE) score < 20/30 (41).

### 2.3 Data collection and study variables

Data collection followed a standardized protocol performed by trained personnel to ensure consistency and reproducibility across all assessments. Sociodemographic, clinical, nutritional, functional, and biochemical variables relevant to aging, cardiometabolic health, and inflammation were collected. The main domains are described below, and further details about the assessment methods, including cut-offs and the related bibliography, can be found in Supplementary Table S1.

Sociodemographic and clinical data. They were collected through structured or semi-structured interviews and included age, sex, ethnicity, educational level, marital status, occupation, housing status, residential area, social support, smoking habits, medication use, previous hospitalizations. Comorbidity burden was assessed through the Cumulative Illness Rating Scale (CIRS).

Anthropometry and body composition. Body weight was measured, while height was estimated using validated predictive methods for older adults based on knee height and demi-span measurements. Body Mass Index (BMI) was calculated based on weight and height derived from demi-span. Circumferences (waist, mid-upper arm, and calf) were measured according to standardized procedures: waist circumference is recognized as an independent predictor of cardiovascular risk, morbidity, and mortality, beyond BMI; mid-upper arm circumference is used as a proxy for malnutrition; and calf circumference is considered a proxy for muscle mass in older populations. Body composition was evaluated through bioelectrical impedance analysis (BIA; BIA-101, Akern, Florence, Italy) that allowed the assessment of phase angle, fat-free mass, fat mass, appendicular skeletal muscle mass (ASM), and body water. ASM was estimated using the equation developed by Sergi et al., specifically validated for the prediction of appendicular skeletal muscle mass from BIA in older European adults. When BIA was not feasible, calf circumference was used to complete the EWGSOP2 diagnostic algorithm, in accordance with the guideline recommendation to adopt it as a diagnostic proxy for muscle mass where direct assessment is unavailable.

Laboratory assessments. Fasting blood samples were analyzed for a wide panel of biochemical markers, encompassing markers of inflammation and metabolic and nutritional status. Circulating cytokines (e.g., IL-1β, IL-6, IL-10, TNF-α, TGF-β) were measured using commercially available kits from R&D Systems. Samples were assayed in duplicate with internal quality control. Missing laboratory data reflected clinical and logistical constraints typical of hospitalized older adults, as well as the fact that serum collection for cytokine measurements was incorporated into the protocol only after recruitment had begun, rather than selective exclusion of participants (see Supplementary materials for details).

Lifestyle assessment. Lifestyle factors were assessed using validated questionnaires. Adherence to healthy dietary patterns was assessed through the MEDI-LITE score and the MIND diet score. Alcohol habits were also investigated through structured interviews. Physical activity was measured through the Physical Activity Scale for the Elderly (PASE), while activities of daily living were investigated through the validated Barthel Index. Sleep quality was assessed through the Pittsburgh Sleep Quality Index (PSQI), health-related quality of life through the Short Form-12 (SF-12), and vitamin D deficiency risk through the EVIDENCe-Q questionnaire.

Physical performance and muscle strength. Muscle strength was evaluated through handgrip dynamometry (DynEX, Akern srl) performed on the non-dominant hand, as neither the EWGSOP2 consensus nor the dynamometer manufacturer prescribes the use of a specific hand. Three consecutive maximal-effort trials were performed under standardized conditions, and the mean value was used; the detailed measurement protocol is reported in Supplementary Table S1. Physical performance was assessed through the 4-meter gait speed test with a static start.

Cognitive, psychological and multidimensional function assessment. Cognitive status was evaluated through the Mini-Mental State Examination (MMSE) as an eligibility criterion, while depressive symptoms were assessed through the 15-item Geriatric Depression Scale (GDS-15).

### 2.4 International standardized diagnostic criteria used

Malnutrition. It was diagnosed according to the Global Leadership Initiative on Malnutrition (GLIM) consensus criteria (3). The validated Malnutrition Universal Screening Tool (MUST) was used to screen malnutrition risk. Malnutrition was diagnosed based on at least one phenotypic and one etiologic criterion. Severity grading was based on the extent of phenotypic alterations. Given the lack of universally accepted cut-offs based on muscle mass parameters, severity was further refined using sex-specific percentile distributions of appendicular skeletal muscle mass (ASM) and calf circumferences, as previously suggested in the literature (42–45). Detailed criteria are reported in Supplementary Table S2.

Sarcopenia. It was defined according to the European Working Group on Sarcopenia in Older People (EWGSOP2) guidelines (4), using a stepwise approach based on low muscle strength (probable sarcopenia), reduced muscle quantity/quality (confirmed diagnosis), and impaired physical performance (severe sarcopenia). Handgrip strength, ASM assessed by BIA, calf circumference, and 4-meter gait speed were used according to recommended cut-offs (Supplementary Table S3).

Sarcopenic obesity. It was identified according to the recently proposed ESPEN/EASO consensus (5). After assessing central obesity and sarcopenia indicators (screening), low muscle strength and altered body composition were investigated to confirm the diagnosis (Supplementary Table S3). Finally, staging was based on the presence of obesity-related clinical complications, including endocrine-metabolic diseases (including hepatic steatosis), cardiovascular diseases, respiratory diseases, neurodegenerative diseases, and functional disability (identified using the CIRS and the Barthel Index).

Metabolic syndrome. The diagnosis was made in accordance with the criteria of the International Diabetes Federation (IDF) (46). It was based on waist circumference above the reference sex-specific cut-offs, and on the presence of at least two comorbidities, including hypertriglyceridemia, reduced HDL cholesterol, hypertension, or elevated plasma glucose or type 2 diabetes. Detailed cutoffs used are reported in Supplementary Table S3.

Cardiometabolic diseases. The diagnosis of cardiovascular and endocrine-metabolic diseases was established or confirmed by physicians within the study team. Afterwards, a physician completed the CIRS for each participant. Cardiovascular diseases were defined by combining the cardiac, hypertension, and vascular disease subscales of the CIRS, whereas endocrine–metabolic diseases were assessed using the endocrine–metabolic subscale, supplemented with the presence of hepatic steatosis. In accordance with the CIRS guidelines, a score of ≥1 on each relevant subscale was considered indicative of the corresponding disease category. Related references are provided in the Supplementary materials.

### 2.5 Statistical analysis

The sample size for the cross-sectional phase was estimated at 200 participants. Assuming a prevalence of malnutrition among Italian older adults (≥ 65 years) ranging between 11 and 22% (47), this number ensures 80% statistical power at a significance level of 0.05. The primary endpoint, namely the prevalence of malnutrition, was expressed as the proportion of participants diagnosed with malnutrition among the total number of subjects enrolled in the study. The same approach was used for the other prevalences. All variables collected during the study (including physical, nutritional, and sociodemographic factors), except those involved in the diagnostic processes, were analyzed according to malnutrition status, sarcopenia and sarcopenic obesity. Exploratory logistic regression analysis was also performed for sarcopenic diabetes.

Data processing, quality checks and statistical analysis were all done using STATA 18 (StataCorp LLC, College Station, TX). Analyses were performed using available-case data without imputation of missing values. Collected data were described using basic descriptive statistics, including means, standard deviations (SD), frequencies and percentages. Group comparisons of continuous variables were performed using parametric or non-parametric tests according to data distribution, whereas discrete variables were compared using Chi-square or Fisher’s exact tests. To correct the *p*-value for multiple comparisons, the Sidak method was applied to all comparisons performed, since it offers a better balance between Type I and Type II errors compared to the Bonferroni method.

For assessing the correlations with inflammatory, nutritional, and metabolic biomarkers, different correlation coefficients were selected according to the distributional properties of the variables under consideration. Specifically, Pearson’s correlation coefficient was applied to continuous variables following a normal distribution, while Spearman’s rank correlation coefficient (a non-parametric alternative) was used for variables deviating from normality or characterized by a high number of outliers.

Associations of malnutrition, sarcopenia, sarcopenic obesity, and sarcopenic diabetes (exploratory) were assessed by logistic and ordered logistic models. For each outcome, candidate variables were selected *a priori* based on clinical knowledge, existing literature, and biological plausibility, considering sociodemographic, clinical, and lifestyle factors previously reported as potentially associated with these outcomes. Sex (males/females), age (according to the Italian SIGG classification) (48), recruitment setting (inpatients/outpatients), disease burden (CIRS severity ≥ 2.5), and the presence of at least one acute disease were included as potential confounders in all models, except where their inclusion would have introduced circularity. Collinearity and interactions among candidate variables were also assessed. Lifestyle variables tested included low adherence to healthy dietary patterns (MEDI-LITE score < 8.5 for the Mediterranean diet; MIND Diet score < 8.5), low physical activity level (PASE < 76), functional dependence (Barthel Index ≤ 70), poor sleep quality (PSQI > 5), low quality of life (PCS ≤ 50 and MCS ≤ 42), and presence of depressive symptoms (GDS-15 > 5); the corresponding cut-offs were derived from the literature (Supplementary Table 1), except for CIRS severity and the MIND diet score, for which the median value of the sample distribution was used in the absence of validated thresholds. The CIRS comorbidity index was excluded from the candidate variables due to its strong correlation with CIRS severity (Pearson’s ρ = 0.94, *p* < 0.0001), reflecting substantial collinearity between the two indices; only CIRS severity was therefore retained in the models.

## 3 Results

### 3.1 Participant characteristics

A total of 303 Caucasian participants were enrolled, including 233 inpatients and 70 outpatients. The complete recruitment flowchart and missing data details are provided in the Supplementary materials. The cohort had a mean age of 76.6 ± 7.3 years, with a balanced sex distribution (49.8% female). Most participants were retired and had a low educational level (49). Compared with outpatients, inpatients were older, differed significantly in age classes (48), and showed different socio-demographic characteristics (Table 1).

**Table 1:** Participant characteristics.

Characteristic		Total	Inpatients	Outpatients	*p*-value
Socio-demographics	Sex				
	Males	152 (50.2)	123 (40.6)	29 (9.6)	0.095
	Females	151 (49.8)	110 (36.3)	41 (13.5)	
	Age (years)	76.6 ± 7.3	78.1 ± 7.2	71.9 ± 5.3	**<0.001**
	Age classes according to SIGG (years)				
	65–74	123 (40.6)	73 (24.1)	50 (16.5)	**<0.001**
	75–84	138 (45.5)	119 (39.3)	19 (6.3)	
	85–99	41 (13.5)	40 (13.2)	1 (0.3)	
	≥100	1 (0.3)	1 (0.3)	0 (0)	
	Education level				
	None	2 (0.7)	2 (0.7)	0 (0)	**0.001**
	Primary school	80 (26.6)	71 (23.6)	9 (3.0)	
	Lower secondary school	95 (31.6)	63 (20.9)	32 (10.6)	
	High school	95 (31.6)	68 (22.6)	27 (9.0)	
	University degree or higher	29 (9.6)	27 (9.0)	2 (0.7)	
	Marital status				
	Married/cohabiting	180 (59.4)	125 (41.3)	55 (18.2)	**0.001**
	Separated	11 (3.6)	9 (3.0)	2 (0.7)	
	Divorced	15 (5.0)	11 (3.6)	4 (1.3)	
	Widowed	79 (26.1)	74 (24.4)	5 (1.7)	
	Single	18 (5.9)	14 (4.6)	4 (1.3)	
	Housing status				
	Institution	4 (1.3)	4 (1.3)	0 (0)	0.055
	Alone	76 (25.3)	62 (20.6)	14 (4.7)	
	With spouse	146 (48.5)	103 (34.2)	43 (14.3)	
	With spouse and children	32 (10.6)	22 (7.3)	10 (3.3)	
	With children	32 (10.6)	30 (10.0)	2 (0.7)	
	With caregivers	5 (1.7)	5 (1.7)	0 (0)	
	With siblings	4 (1.3)	3 (1.0)	1 (0.3)	
	Other relatives	2 (0.7)	2 (0.7)	0 (0)	
	Social support				
	None	18 (5.9)	13 (4.3)	5 (1.7)	0.415
	Siblings	47 (15.6)	32 (10.6)	15 (4.9)	
	Sons	194 (64.4)	152 (50.5)	42 (13.9)	
	Nephews	13 (4.3)	10 (3.3)	3 (1.0)	
	Other	29 (9.6)	25 (8.3)	4 (1.3)	
	Residential area				
	Pavia	54 (18.1)	47 (15.7)	7 (2.3)	**0.007**
	Province of Pavia	179 (59.9)	124 (41.5)	55 (18.4)	
	Lombardy	47 (15.7)	40 (13.4)	7 (2.3)	
	Other regions	18 (6.0)	17 (5.7)	1 (0.3)	
	Not reported	1 (0.3)	1 (0.3)	0 (0)	
	Occupation				
	Employed	24 (7.9)	19 (6.3)	5 (1.7)	0.783
	Retired	279 (92.1)	214 (70.6)	65 (21.5)	
Clinical variables	CIRS, severity index	2.4 ± 0.5	2.6 ± 0.4	1.9 ± 0.4	**<0.001**
	CIRS, comorbidity index	6.6 ± 2.4	7.3 ± 1.9	4.1 ± 2.2	**<0.001**
	Polypharmacy				
	< 5 drugs taken	71 (23.4)	40 (13.2)	31 (10.2)	**<0.001**
	≥5 drugs taken	232 (76.6)	193 (63.7)	39 (12.9)	
	Hospitalizations in the previous year				
	No	197 (65.0)	144 (47.5)	53 (17.5)	**0.032**
	Yes	106 (35.0)	89 (29.4)	17 (5.6)	
Anthropometry and body composition	Body weight (kg)	74.9 ± 19.3	76.1 ± 20.3	70.9 ± 14.7	**0.044**
	Height derived from demi-span (cm)	163.4 ± 7.7	163.2 ± 7.5	164.0 ± 8.4	0.449
	Height derived from knee height (cm)	160.6 ± 8.2	160.3 ± 8.2	161.7 ± 8.0	0.199
	BMI (kg/m^2^)	28.0 ± 6.6	28.5 ± 7.1	26.2 ± 4.3	**0.010**
	Waist circumference (cm)	103.9 ± 16.2	106.3 ± 16.2	96.3 ± 14.2	**<0.001**
	Mid-arm circumference (cm)	29.5 ± 5.7	29.3 ± 5.5	30.2 ± 6.4	0.308
	Calf circumference (cm)	35.4 ± 4.9	35.1 ± 5.4	36.2 ± 3.3	0.127
	Fat-free mass (kg)	51.8 ± 11.4	51.6 ± 11.4	52.4 ± 11.2	0.605
	Fat mass (kg)	23.1 ± 12.1	25.0 ± 13.0	18.3 ± 7.8	**<0.001**
	Phase angle (°)	4.7 ± 0.8	4.5 ± 0.8	5.4 ± 0.6	**<0.001**
	ASM (kg)	18.6 ± 4.7	18.6 ± 4.9	18.6 ± 4.2	0.985
	ASMI (kg/m^2^)	6.9 ± 1.4	6.9 ± 1.6	6.9 ± 1.1	0.735
Lifestyle	Adherence to Mediterranean diet (MEDI-LITE score)	11.5 ± 2.0	11.4 ± 2.1	11.7 ± 1.9	0.274
	Adherence to MIND diet (MIND diet score)	8.3 ± 1.6	8.3 ± 1.6	8.5 ± 1.5	0.263
	Alcohol consumption: wine, beer, aperitifs				
	Never	13 (5.9)	9 (4.1)	4 (1.8)	0.551
	Occasional (< 1 portion/week)	74 (33.6)	52 (23.6)	22 (10.0)	
	Habitual (≥1 portion/week)	133 (60.5)	102 (46.4)	31 (14.1)	
	Alcohol consumption: digestifs, spirits				
	Never	88 (40.2)	63 (28.8)	25 (11.4)	0.455
	Occasional (< 1 portion/week)	106 (48.4)	79 (36.1)	27 (12.3)	
	Habitual (≥1 portion/week)	25 (11.4)	21 (9.6)	4 (1.8)	
	Smoking habits				
	Never	124 (40.9)	100 (33.0)	24 (7.9)	0.425
	Former smoker	138 (45.5)	102 (33.7)	36 (11.9)	
	Current smoker	41 (13.5)	31 (10.2)	10 (3.3)	
	Physical activity level (PASE)	91.3 ± 57.2	76.4 ± 52.3	140.3 ± 44.1	**<0.001**
	Activities of daily living (Barthel Index)	87.7 ± 18.4	84.8 ± 20	97.5 ± 4.6	<0.001*
	Sleep quality (PSQI score)	8.0 ± 3.8	8.4 ± 3.8	6.6 ± 3.5	**<0.001**
	Quality of life (SF-12)				
	PCS-12 score	38.9 ± 11.1	36.4 ± 10.7	47.2 ± 8.3	**<0.001**
	MCS-12 score	48.3 ± 10.5	47.1 ± 10.9	52.0 ± 8.5	**<0.001**
	Depressive symptoms (GDS-15)	4.7 ± 3.3	5.3 ± 3.3	3.0 ± 2.6	**<0.001**
	Vitamin D deficiency (EvidenceQ)	20.8 ± 3.7	21.7 ± 3.6	18.9 ± 3.1	<0.001*
Functional assessment	Handgrip strength (kg)	18.0 ± 7.7	17.2 ± 7.6	20.6 ± 7.6	**0.001**
	Locomotion through 4-m gait speed (m/s)	0.8 ± 0.4	0.7 ± 0.3	1.0 ± 0.4	<0.001*

Data are expressed as mean ± standard deviation, or number (percentage). *p*-values were derived using the chi-square test or Student’s t-test; the Mann–Whitney U test was applied where indicated (*). ASM, Appendicular skeletal mass; ASMI, Appendicular skeletal mass index; BMI, Body Mass Index; CIRS, Cumulative Illness Rating Scale; EvidenceQ, Evaluation VItamin D dEficieNCy Questionnaire; GDS-15, Geriatric Depression Scale 15 items; MCS-12, Mental Component Summary; MIND diet, Mediterranean-DASH Intervention for Neurodegenerative Delay diet; PASE, Physical Activity Scale for the Elderly; PCS-12, Physical Component Summary; PSQI, Pittsburgh sleep quality index; SIGG, Società Italiana di Gerontologia e Geriatria; SF-12, Short-Form Health Survey 12 items. *p*-values were adjusted for multiple comparisons using the Sidak correction, with statistically significant values highlighted in bold.

The study population exhibited a moderate-to-high clinical burden, characterized by the co-occurrence of multiple chronic diseases in the same individuals (mean CIRS comorbidity index: 6.6 ± 2.4) and a high prevalence of polypharmacy (76.6%; 95% CI: 71.4–81.2) defined as patients taking more than 5 drugs chronically. Inpatients showed a worse clinical profile than outpatients, with higher comorbidity indices, greater polypharmacy, and more frequent previous hospitalization.

Anthropometric and body composition analyses revealed a high prevalence of overweight (30.7%; 95% CI: 25.5–36.2%) and obesity (33.3%; 95% CI: 28–38.9%) in the total sample, with a mean waist circumference (103.9 ± 16.2 cm), which suggested increased cardiovascular risk (46). Among patients with obesity, 57.4% had class I, 31.7% class II and 10.9% class III obesity. Instead, the prevalence of underweight was low (4.6%; 95% CI: 2.5–7.6%). Mean phase angle (4.7 ± 0.8°) fell below the reference values for those over 60 (50), suggesting impaired cell membrane integrity and suboptimal nutritional status. Hospitalized patients had higher body weight, BMI, waist circumference, and fat mass than outpatients, while showing lower handgrip strength, gait speed, and phase angle.

With reference to lifestyle, participants showed moderate adherence to both the Mediterranean (mean score: 11.5 ± 2) and MIND diets (8.3 ± 1.6), alongside low physical activity levels (mean PASE score: 91.3), which was, however, above the insufficiency threshold of 76 applied in the Italian national surveillance system Passi d’Argento for older adults (51). Compared to outpatients, hospitalized patients exhibited lower physical activity levels, greater dependence in performing activities of daily living, poorer sleep quality and health-related quality of life, as well as higher prevalence of depressive symptoms and suspected vitamin D deficiency.

### 3.2 Primary findings

#### 3.2.1 Prevalence of malnutrition, sarcopenia, and sarcopenic obesity

In the total sample, the prevalence of malnutrition, sarcopenia, and sarcopenic obesity was 39.9%, 30.9%, and 9.4%, respectively (Table 2). A high prevalence of probable sarcopenia (42.6%) and malnutrition risk (medium 33.9% or high 24.6%) was also retrieved. Notably, malnutrition coexisted with overweight/obesity in 13.2% of enrolled participants. Among patients diagnosed with malnutrition via GLIM, 56.2% had a normal weight, 33.0% were overweight or obese (24.0 and 9.0%, respectively), and 10.7% were underweight.

**Table 2:** Prevalence of malnutrition, sarcopenia, and sarcopenic obesity.

Screening and diagnostic criteria		Total	Inpatients	Outpatients	*p*-value
Risk of malnutrition (MUST)	Low risk (0 points)	125 (41.5; 95% CI: 35.9–47.3)	62 (26.7)	63 (91.3)	**<0.001**
	Medium risk (1 point)	102 (33.9; 95% CI: 28.6–39.5)	100 (43.1)	2 (2.9)	
	High risk (≥2 points)	74 (24.6; 95% CI: 19.8–29.9)	70 (30.2)	4 (5.8)	
Malnutrition (GLIM)	No malnutrition	182 (60.1)	123 (52.8)	59 (84.3)	**<0.001**
	Mild/moderate malnutrition	74 (24.4; 95% CI: 19.7–29.7)	66 (28.3)	8 (11.4)	
	Severe malnutrition	47 (15.5; 95% CI: 11.6–20.1)	44 (18.9)	3 (4.3)	
Sarcopenia^a^ (EWGSOP2)	No sarcopenia	79 (26.5)	43 (18.8)	36 (52.2)	**<0.001**
	Probable sarcopenia	127 (42.6; 95% CI: 36.9–48.4)	104 (45.4)	23 (33.3)	
	Confirmed sarcopenia	53 (17.8; 95% CI: 13.6–22.6)	46 (20.1)	7 (10.1)	
	Severe sarcopenia	39 (13.1; 95% CI: 9.5–17.5)	36 (15.7)	3 (4.3)	
Sarcopenic obesity (ESPEN/ EASO)^b^	No sarcopenic obesity	113 (37.9)	75 (32.8)	38 (55.0)	**<0.001**
	Probable sarcopenic obesity	157 (52.7; 95% CI:46.8–58.5)	126 (55.0)	31 (44.9)	
	Confirmed sarcopenic obesity (Stage I)	0 (0)	0 (0)	0 (0)	
	Severe sarcopenic obesity (Stage II)	28 (9.4; 95% CI: 6.3–13.3)	28 (12.2)	0 (0)	

a Of 303 participants for which sarcopenia was assessed, 5 had missing handgrip strength data and were excluded from this analysis (*n* = 298, presented in the table). Among these, 13 participants had neither BIA nor calf circumference available and could therefore not receive a confirmed diagnosis; they are included in the “probable sarcopenia” category.

b Of 298 participants with available handgrip strength data, 157 met criteria for probable sarcopenic obesity; among these, 42 could not be confirmed due to missing BIA data (*n* = 62 missing overall, primarily due to contraindications to its use), as the ESPEN/EASO consensus does not permit the use of calf circumference as a substitute for BIA when body composition data are unavailable. A sensitivity analysis restricted to the 237 participants with complete handgrip strength and BIA data (303–5–61 = 237, since one participant lacked both handgrip and BIA) is presented in the Supplementary materials.

Data are expressed as number (percentage relative to the column totals); 95% confidence intervals are provided for the total sample. *p*-values were derived using the chi-square test, with statistically significant values highlighted in bold. ESPEN/EASO, European Society for Clinical Nutrition and Metabolism/European Association for the Study of Obesity; EWGSOP2, European Working Group on Sarcopenia in Older People 2; GLIM, Global Leadership Initiative on Malnutrition; MUST, Malnutrition Universal Screening Tool. *p*-values were adjusted for multiple comparisons using the Sidak correction. *N* = 301 for malnutrition risk, *n* = 303 for malnutrition, *n* = 298 for sarcopenia, *n* = 298 for sarcopenic obesity.

Marked differences in all the prevalences emerged between settings (*p* < 0.001). Hospitalized patients exhibited a substantially higher burden of malnutrition risk and diagnosis compared to outpatients. Similarly, probable and confirmed sarcopenia were significantly more prevalent among inpatients. Among 298 participants with handgrip strength results available, only 13 participants (4.4%) had both BIA and calf circumference missing; given this small proportion, a sensitivity analysis was not deemed necessary. Locomotion assessment was limited in the inpatient setting, where 27.4% of participants were unable to perform this test due to being bedridden, having an unstable gait or a high risk of falls.

Sarcopenic obesity was observed in 12.2% of inpatients, all classified as Stage II severity, whereas no cases were identified among outpatients. Probable sarcopenic obesity was more frequent (55 and 44.9%, respectively). However, unlike the EWGSOP2 algorithm for sarcopenia, calf circumference could not be used as a substitute for BIA to complete this diagnosis. Consequently, confirmation was precluded in 42 participants with probable sarcopenic obesity due to missing BIA measurements (*n* = 62 missing overall, primarily due to contraindications to its use). Thus, a subanalysis is presented in the Supplementary materials to assess the prevalence of sarcopenic obesity among the 237 participants for whom all variables required for diagnosis were available (303–5–61 = 237, since one participant lacked both handgrip and BIA).

The overlap of conditions was frequent, particularly in inpatients. The coexistence of malnutrition with sarcopenia or probable sarcopenia reached 29.2 and 35.9%, respectively. The simultaneous presence of malnutrition, sarcopenia, and sarcopenic obesity was rare (2.0% overall; 2.7% in inpatients). Conversely, the combination of malnutrition, sarcopenia, and *probable* sarcopenic obesity was highly prevalent, affecting 20.2% of the total population (24.8% of inpatients vs. 5.8% of outpatients).

#### 3.2.2 Factors associated with malnutrition, sarcopenia and sarcopenic obesity

Logistic regression analysis showed that older adults aged 75–84 years and the oldest-old participants (85–99 years) exhibited significantly greater odds of malnutrition: OR = 2.20 (95% CI: 1.21–4.00; *p* = 0.010) and OR = 8.53 (95% CI: 3.27–22.27; *p* < 0.001), respectively. Higher odds were also observed for individuals reporting poor physical health (OR = 3.17; 95% CI: 1.39–7.22; *p* = 0.006; Table 3). Notably, due to a moderate correlation between PASE and Barthel scores (*r* = 0.45), only the latter was included in the model. To avoid circularity, acute disease and BMI were not entered into the present logistic regression model, as both are included among the criteria used to diagnose malnutrition according to the GLIM framework.

**Table 3:** Multivariable logistic regression model of factors associated with malnutrition, sarcopenia or sarcopenic obesity.

Diagnostic criteria	Factors	Odds ratio (95% CI)	Std. err.	z	*p*-value
Malnutrition (GLIM)	Age (SIGG age classes)				
	75–84 years	2.20 (1.21–4.00)	0.67	2.59	**0.010**
	85–99 years	8.53 (3.27–22.27)	4.18	4.38	**< 0.001**
	Dependence in activities of daily living (Barthel Index ≤ 70)	2.05 (0.87–4.81)	0.89	1.65	0.099
	Poor physical health-related quality of life (PCS-12 ≤ 50, part of SF-12)	3.17 (1.39–7.22)	1.33	2.75	**0.006**
	Setting (inpatients, outpatients)	0.59 (0.26–1.32)	0.24	−1.29	0.197
	Disease burden (CIRS severity index ≥2.5)	1.44 (0.80–2.59)	0.43	1.20	0.229
Sarcopenia (EWGSOP2)	Age (SIGG age classes)				
	75–84 years	1.70 (0.73–3.97)	0.74	1.23	0.220
	85–99 years	2.23 (0.67–7.37)	1.36	1.31	0.189
	Dependence in activities of daily living (Barthel Index ≤ 70)	5.39 (1.68–17.35)	3.22	2.83	**0.005**
	Poor physical health-related quality of life (PCS-12 ≤ 50, part of SF-12)	3.90 (1.31–11.62)	2.17	2.44	**0.015**
	Setting (inpatients, outpatients)	0.35 (0.10–1.16)	0.21	−1.72	0.086
	Disease burden (CIRS severity index ≥2.5)	0.76 (0.33–1.72)	0.32	−0.66	0.508
	Acute diseases	1.15 (0.44–2.98)	0.56	0.28	0.779
	BMI class (WHO)				
	Underweight (BMI < 18.5 kg/m^2^)	3.06 (0.54–17.23)	2.70	1.27	0.204
	Overweight (BMI: 25–29.9 kg/m^2^)	0.18 (0.08–0.40)	0.07	−4.28	**< 0.001**
	Obesity (BMI ≥ 30 kg/m^2^)	0.02 (0.00–0.06)	0.01	−6.03	**< 0.001**
Sarcopenic obesity (ESPEN/ EASO)	Age (SIGG age classes)				
	75–84 years	2.00 (1.14–3.50)	0.57	2.42	**0.016**
	85–99 years	1.01 (0.44–2.28)	0.42	0.02	0.985
	Low physical activity level (PASE < 76)	1.80 (1.02–3.18)	0.52	2.04	**0.041**
	Setting (inpatients, outpatients)	0.54 (0.26–1.10)	0.20	−1.70	0.089
	Disease burden (CIRS severity index ≥2.5)	1.10 (0.61–1.98)	0.33	0.33	0.743
	Acute diseases	0.91 (0.48–1.70)	0.29	−0.30	0.763

Odds ratios (ORs), 95% confidence intervals (95% CIs), standard errors, z, and *p*-values are reported, with statistically significant *p* values highlighted in bold. As the centenarian age category comprised only one participant, it could not be reliably included as a level of the age-class variable and was therefore excluded from the model.

When logistic regression analysis was applied to sarcopenia, dependent participants (OR = 5.39; 95% CI: 1.68–17.35; *p* = 0.005) and those reporting poor physical health (OR = 3.90; 95% CI: 1.31–11.62; *p* = 0.015) were also at markedly increased risk. Instead, overweight and obesity were associated with significantly lower odds of confirmed sarcopenia: OR = 0.18 (95% CI: 0.08–0.40; *p* < 0.001) and OR = 0.02 (95% CI: 0.00–0.06; *p* < 0.001), respectively (Table 3).

Logistic regression analysis showed that participants aged 75–84 years (OR = 2.00; 95% CI: 1.14–3.50; *p* = 0.016) and low physical activity levels (OR = 1.80; 95% CI: 1.02–3.18; *p* = 0.041) had a higher likelihood of having sarcopenic obesity (Table 3). A subanalysis (Supplementary materials) was also conducted using logistic regression models to assess factors independently associated with sarcopenic obesity among the 237 participants for whom all variables required for diagnosis were available, stratified by inpatients (no confirmed outpatient cases were available for an outpatient model).

### 3.3 Secondary findings

#### 3.3.1 Prevalence of metabolic syndrome and cardiometabolic diseases

Overall, the vast majority of participants suffered from metabolic syndrome (60.1%; 95% CI: 54.3–65.6%), cardiovascular diseases (96%; 95% CI: 93.2–97.9%) and endocrine-metabolic diseases (97.4%; 95% CI: 94.8–98.8%; Table 4). Metabolic syndrome was more prevalent in inpatients (67%) than in outpatients (37.1%). Similarly, cardiovascular diseases were more frequently observed in hospitalized patients (99.1%) than in free-living older adults (85.5%). Notably, the prevalence of endocrine-metabolic diseases was comparable between groups (97.4% vs. 97.1%).

**Table 4:** Prevalence of metabolic syndrome and cardiometabolic diseases.

Cardiometabolic health and diseases		Total	Inpatients	Outpatients	*p*-value
Metabolic syndrome (IDF)	No metabolic syndrome	121 (39.9)	77 (33.0)	44 (62.9)	**<0.001**
	Metabolic syndrome	182 (60.1; 95% CI: 54.3–65.6)	156 (67.0)	26 (37.1)	
Cardiometabolic diseases	Cardiovascular diseases (≥1 diagnosed)				
	No	12 (4.0)	2 (0.9)	10 (14.5)	**<0.001**
	Si	290 (96.0; 95% CI: 93.2–97.9)	231 (99.1)	59 (85.5)	
	Metabolic diseases (≥1 diagnosed)				
	No	8 (2.7)	6 (2.6)	2 (2.9)	0.883
	Si	294 (97.4; 95% CI: 94.8–98.8)	227 (97.4)	67 (97.1)	

Data are expressed as number (percentage relative to the column totals); 95% confidence intervals are provided for the total sample. *p*-values were derived using the chi-square test, with statistically significant values highlighted in bold. IDF, International Diabetes Federation. *p*-values were adjusted for multiple comparisons using the Sidak correction. *N* = 303 for metabolic syndrome, *n* = 302 for cardiovascular diseases and for metabolic diseases.

The prevalence of metabolic syndrome was significantly higher among non-malnourished than malnourished participants (67.0% vs. 49.6%, *p* = 0.002). Similarly, metabolic syndrome was more prevalent in non-sarcopenic than sarcopenic older adults (65.6% vs. 50.0%, *p* = 0.011). Furthermore, the prevalence of metabolic syndrome differed significantly across participants without sarcopenic obesity, those with probable sarcopenic obesity, and those with confirmed sarcopenic obesity (44.2% vs. 69.4% vs. 75.0%, *p* < 0.01).

The coexistence of malnutrition and metabolic syndrome was observed in 19.8% of the sample, exclusively among hospitalized patients. Similarly, metabolic syndrome co-occurred with sarcopenia in 16% of the participants, and with sarcopenic obesity in 7% of the study sample.

As an exploratory analysis, the coexistence of sarcopenia and type 2 diabetes, an under-recognized clinical priority in clinical nutrition (26), was also assessed. It was observed in 9.6% (95% CI: 6.6–13.4%) of patients; it was not reported among outpatients. Logistic regression analysis showed that patients with a CIRS severity index ≥ 2.5 had substantially increased odds of having sarcopenic diabetes (OR = 10.55; 95% CI: 2.33–47.75; *p* = 0.002; Supplementary materials). The logistic regression model for sarcopenic diabetes stratified by inpatients (no outpatient cases were observed) is also presented in the Supplementary materials.

#### 3.3.2 Setting- and age-related differences in inflammatory, nutritional, and metabolic biomarkers

Compared with outpatients, inpatients exhibited a more pronounced pro-inflammatory profile (Table 5), with significantly higher levels of CRP (4.7 ± 8.6 vs. 0.3 ± 0.4 mg/dL), IL-6 (14.6 ± 22.2 vs. 5.9 ± 16.2 pg./mL), IL-10 (20.4 ± 90.3 vs. 1.4 ± 4.0 pg./mL), white blood cells (8.0 ± 2.9 vs. 6.6 ± 1.9 × 10^9^/L), and uric acid (6.0 ± 2.2 vs. 5.0 ± 1.5 mg/dL), and significantly lower prealbumin (18.3 ± 6.5 vs. 26.5 ± 4.8 mg/dL). Age was significantly associated with CRP, prealbumin, and two cytokines (IL-6 and IL-10; Table 6). Spearman correlation analysis demonstrated a positive correlation between age and IL-10 (ρ = 0.26, *p* < 0.05), IL-6 (ρ = 0.43, *p* < 0.05), and TNF-α (ρ = 0.16, *p* < 0.05), indicating a progressive increase in both pro- and anti-inflammatory mediators with advancing age. Conversely, TGF-β concentrations showed a weak but significant inverse Pearson correlation with age (ρ = −0.16, *p* < 0.05). Additionally, the Supplementary materials include a table comparing inflammatory, nutritional, and metabolic biomarkers levels between males and females. Due to missing values and the high variability observed in the sample, these results should be interpreted with caution.

**Table 5:** Inflammatory biomarker levels according to clinical setting (inpatients vs. outpatients).

Biomarkers	Total		Inpatients		Outpatients		*p*-value
	n	mean (SD)	n (%)	mean (SD)	n (%)	mean (SD)	
C-reactive protein, CRP (mg/dL)	198	3.1 (7.2)	128 (64.6)	4.7 (8.6)	70 (35.4)	0.3 (0.4)	< 0.001*
White blood cell count (109/L)	300	7.7 (2.8)	230 (76.6)	8 (2.9)	70 (23.4)	6.6 (1.9)	< 0.001*
Platelet count (109/L)	298	224.8 (73.2)	228 (76.5)	223.2 (78.3)	70 (23.5)	230.2 (53.8)	0.488
Prealbumin (mg/dL)	165	21.8 (7.1)	95 (57.6)	18.3 (6.5)	70 (42.4)	26.5 (4.8)	**< 0.001**
Uric acid (mg/dL)	151	5.5 (1.9)	81 (53.6)	6.0 (2.2)	70 (46.4)	5.0 (1.5)	**0.002**
Interleukin-6, IL-6 (pg/mL)	212	11.8 (20.8)	143 (67.4)	14.6 (22.2)	69 (32.6)	5.9 (16.2)	< 0.001*
Tumor necrosis factor-α, TNF-α (pg/mL)	211	2.1 (8.3)	142 (67.3)	2.3 (8.6)	69 (32.7)	1.7 (7.8)	0.503
Transforming growth factor-β, TGF-β (pg/mL)	201	25,715 (14595.5)	135 (67.2)	24989.3 (14569.1)	66 (32.8)	27199.6 (14647.7)	0.314
Interleukin-10, IL-10 (pg/mL)	195	14.2 (74.5)	131 (67.2)	20.4 (90.3)	64 (32.8)	1.4 (4.0)	**< 0.001**

Data are presented as mean (standard deviation). For each biomarker, n indicates the number of participants with available data, and percentages indicate the proportion of inpatients and outpatients for whom that biomarker was available. *p*-values were derived using parametric tests, except where indicated (*), in which case non-parametric test (Mann–Whitney) was used. Statistically significant *p*-values are shown in bold. *p*-values were adjusted for multiple comparisons using the Sidak correction.

**Table 6:** Inflammatory and laboratory biomarkers by age group.

Biomarkers	Young-old		Old		Oldest-old		*p*-value
	n	mean (SD)	n	mean (SD)	n	mean (SD)	
CRP (mg/dL)	81	1.6 (4.4)	87	3.0 (5.8)	29	7.8 (13.3)	**<0.001**
WBC (10^9^/L)	122	7.5 (2.8)	136	7.7 (2.9)	41	8.2 (2.6)	0.264
Platelets (10^9^/L)	121	231.8 (61.3)	136	217.2 (81.1)	40	232.2 (76.7)	0.227
Prealbumin (mg/dL)	72	24.9 (6.2)	66	21.1 (6.8)	26	15.4 (5.5)	**<0.001**
Uric acid (mg/dL)	69	5.0 (1.8)	62	5.9 (1.9)	19	6.0 (2.0)	**0.006**
IL-10 (pg/mL)	89	6.3 (28.1)	83	23.4 (110.0)	22	11.3 (9.8)	<0.001*
IL-6 (pg/mL)	95	5.6 (9.9)	89	13.9 (23.9)	27	26.0 (28.8)	<0.001*
TNF-α (pg/mL)	94	0.9 (4.9)	89	2.6 (10.1)	27	4.7 (11.0)	0.028*
TGF-β (pg/mL)	91	27426.9 (15331.6)	85	26169.9 (14080.0)	24	18003.8 (11417.7)	**0.017**

Data are expressed as mean (standard deviation); n indicates the number of participants with available data for each biomarker. *p*-values were derived using parametric tests, except where indicated (*), for which non-parametric tests (Mann–Whitney or Kruskal–Wallis) were used. Age groups were defined as young-old (64–74 years old), old (75–84 years old), and oldest-old (≥85 years old). CRP, C-reactive protein; IL-6, interleukin-6; IL-10, interleukin-10; TGF-β, transforming growth factor-beta; TNF-α, tumor necrosis factor-alpha; WBC, white blood cell count. Statistically significant *p*-values are shown in bold. *p*-values were adjusted for multiple comparisons using the Sidak correction.

#### 3.3.3 Inflammatory, nutritional, and metabolic biomarkers across metabolic syndrome and sarcopenic obesity

Participants with metabolic syndrome displayed evidence of increased systemic inflammation (Supplementary materials). Compared with those without metabolic syndrome, they showed higher CRP concentrations (4.0 ± 8.6 vs. 1.9 ± 4.6 mg/dL, *p* < 0.05), higher white blood cell counts (8.1 ± 2.9 vs. 7.1 ± 2.6 × 10^9^/L, *p* < 0.05), higher uric acid levels (6.0 ± 2.0 vs. 5.1 ± 1.8 mg/dL, *p* < 0.05), and increased circulating IL-6 concentrations (13.2 ± 21.9 vs. 9.8 ± 19.0 pg./mL, *p* < 0.05). Lastly, participants with sarcopenic obesity exhibited a less favorable inflammatory profile compared with those without sarcopenic obesity (Supplementary materials). Specifically, they showed lower prealbumin concentrations (19.2 ± 6.9 vs. 22.6 ± 7.0 mg/dL, *p* < 0.05) and higher circulating IL-6 levels (18.1 ± 23.3 vs. 10.1 ± 19.8 pg./mL, *p* < 0.05). These findings should be interpreted with caution owing to missing data and the potential influence of care setting and disease severity on this association.

#### 3.3.4 Dietary patterns and inflammatory, nutritional, and metabolic biomarkers

Inflammatory, nutritional, and metabolic biomarkers were analyzed according to adherence to the Mediterranean and MIND dietary patterns (Supplementary materials). Participants with high adherence to the Mediterranean diet (MEDI-LITE score ≥8.5) showed significantly lower white blood cell counts compared with those with lower adherence (7.6 ± 2.6 vs. 9.3 ± 5.0 × 10^9^/L, *p* = 0.024). No significant differences were observed for CRP, platelet count, prealbumin, uric acid, or circulating cytokine concentrations, including IL-6, IL-10, TNF-α, and TGF-β. Participants with low adherence to the MIND diet exhibited higher serum uric acid concentrations compared with those with moderate or high adherence (*p* < 0.05), whereas CRP, white blood cell count, platelet count, IL-6, IL-10, TNF-α, and TGF-β levels were similar across adherence categories. Alcohol consumption habits were not significantly associated with circulating inflammatory, nutritional, and metabolic biomarkers analyzed (Supplementary materials). Given the missing values for inflammatory, nutritional, and metabolic biomarkers measurements, caution is warranted when interpreting these results.

## 4 Discussion

The principal finding was the high prevalence and frequent overlap of malnutrition, sarcopenia, sarcopenic obesity and metabolic syndrome in older adults with cardiometabolic diseases, particularly among hospitalized patients, together with their associations with functional and cardiometabolic burden. This observation, in the context of a population characterized by a high prevalence of overweight and obesity, underscores the limitations of BMI alone in assessing nutritional status in older adults. Unlike previous studies, which have generally focused on individual geriatric syndromes or specific metabolic outcomes, our study provides an integrated phenotyping approach. Analyses of inflammatory, nutritional, and metabolic biomarkers and their relationships with adherence to healthy dietary patterns, together with analyses of sarcopenic diabetes, should be considered secondary exploratory findings.

The prevalence of GLIM-defined malnutrition among hospitalized patients (47.2%) was consistent with previous North-Italian and international evidence (8, 52) Although less frequent among endocrinology outpatients (15.7%), malnutrition prevalence remained clinically relevant, suggesting that it may be underrecognized in autonomous older adults with endocrine-metabolic disorders. Similarly, the high prevalence of malnutrition risk (73.2%) in hospitalized patients, and the coexistence of overweight or obesity in one-third of malnourished inpatients further support the concept of malnutrition in obesity (53), underscoring that elevated BMI does not exclude nutritional impairment.

EWGSOP2-defined sarcopenia and probable sarcopenia were also highly prevalent in both settings, especially among inpatients, exceeding previously reported estimates (11, 54). These findings likely reflect differences in settings, study population and methodology (use of calf circumference when BIA was not available). Reduced muscle strength was common even in participants with preserved or increased body weight, whereas the inability to assess gait speed in 27% of the participants, similar to previous Italian studies (55, 56), reflects the clinical instability and frailty of the inpatient population. It should be considered when interpreting estimates of severe sarcopenia.

Sarcopenic obesity was observed only among hospitalized patients (12.2%), with a prevalence in line with previous Italian studies but lower than international data (57, 58), whereas probable sarcopenic obesity was frequent in both settings. The absence of confirmed cases among outpatients may be due to the smaller sample size, younger age, lower disease burden, and missing body composition data. Nonetheless, the subanalysis restricted to the 237 participants for whom all variables required for diagnosis were available showed a similar prevalence of sarcopenic obesity to that observed in the overall sample.

The overlap between malnutrition and sarcopenia (29.2%), previously reported (6), suggests the presence of shared determinants, such as reduced dietary intake, inflammation, disease burden, physical inactivity, and hospital-associated deconditioning. Similarly, the coexistence of sarcopenia and sarcopenic obesity (2%) is clinically plausible (59). Although the coexistence of all three conditions was uncommon (3%), the frequent overlap involving probable sarcopenic obesity (20.2%) suggests the presence of an early high-risk phenotype warranting clinical attention.

Advanced age, and poorer physical health-related quality of life were independently associated with malnutrition. This is consistent with previous evidence identifying advanced age and poor self-perceived health as determinants of malnutrition (60, 61). Instead, functional dependence was independently associated with sarcopenia, as previously reported (62, 63). Poorer physical health-related quality of life was also independently associated with sarcopenia, in line with the relationship between sarcopenia and impaired physical functioning. These factors may act both as determinants and consequences of nutritional and muscle impairment, contributing to a cycle of disability and reduced wellbeing (64). Furthermore, overweight and obesity were associated with lower odds of sarcopenia, suggesting limitations of current diagnostic criteria in individuals with excess adiposity, as well as possible confounding factors (65). Sarcopenic obesity was independently associated with age 75–84 years and low physical activity levels, supporting the role of age-related body composition changes and sedentary behavior in this phenotype (5). Although included in the models as potential confounders, setting, disease burden severity (CIRS), and acute disease were not significantly associated with any of the three outcomes (malnutrition, sarcopenia, and sarcopenic obesity).

Metabolic syndrome was highly prevalent, particularly among inpatients (67%), exceeding previous European estimate (66), while its prevalence in outpatients (37.1%) was consistent with previous Italian evidence (67). The higher prevalence observed among hospitalized patients likely reflects the greater burden of obesity, central adiposity, and multimorbidity in this setting. Notably, metabolic syndrome was significantly associated with malnutrition, sarcopenia, and sarcopenic obesity, supporting the close interplay between metabolic and geriatric syndromes. The prevalence of coexisting sarcopenic obesity and metabolic syndrome observed in our sample (7%) falls within the wide range reported across studies, which varies considerably depending on the diagnostic criteria used to define sarcopenic obesity, the population setting, and whether prevalence is calculated relative to the total sample or conditional on the presence of sarcopenic obesity (68, 69).

As an exploratory analysis, the coexistence of sarcopenia and type 2 diabetes was observed in 9.6% of patients, which was higher than Italian estimates (70). Sarcopenic diabetes was independently associated with greater disease burden (Supplementary materials). It is biologically plausible, as diabetes-related complications and comorbidities have been associated with a higher prevalence of sarcopenia (24).

The inflammatory profile observed in this cohort is consistent with the concept of inflammaging (16). Higher IL-6 concentrations were associated with advancing age, sarcopenic obesity and metabolic syndrome, consistent with the established role of IL-6 as a principal biomarker of inflammaging and its association with muscle loss, and metabolic dysfunction in older adults (71, 72). Given the cross-sectional design, however, these exploratory observations do not imply causal relationships, warranting confirmation through longitudinal studies. Conversely, IL-10, TNF-α and TGF-β showed less consistent association, potentially reflecting the complex regulation of circulating cytokines by ageing, nutritional status, and other biological factors (16). While their biological relevance is well established, their value as circulating biomarkers of cardiometabolic burden appears weaker than that of IL-6.

Beyond circulating cytokines, the additional laboratory biomarkers provided complementary information on nutritional and metabolic status. Lower prealbumin concentrations were observed in participants with sarcopenic obesity and may reflect poorer nutritional status (31, 32). However, as a negative acute-phase reactant, prealbumin concentrations may also be influenced by systemic inflammation (32). Moreover, metabolic syndrome was associated with higher CRP, white blood cell count, and uric acid levels, consistent with a greater inflammatory and metabolic burden (33, 73). The markedly higher levels of CRP and IL-6, together with neutrophilia, among inpatients suggest a greater degree of systemic inflammatory activation compared with outpatients, while the concomitant increase in IL-10 may reflect a counter-regulatory response to inflammation. This pattern may be related to the acute clinical conditions and greater disease burden represented in the inpatient setting.

With reference to dietary patterns, higher adherence to the Mediterranean diet was associated with lower white blood cell counts, whereas lower adherence to the MIND diet was associated with higher serum uric acid concentrations. Due to the heterogeneous evidence on the anti-inflammatory effects of the Mediterranean diet in older populations (74) and the cross-sectional design, no conclusions can be drawn regarding the direction or causality of the observed association. It might reflect a modest, systemic inflammatory signal. Similarly, uric acid may reflect greater metabolic-inflammatory burden, as it is associated with insulin resistance, adiposity and cardiometabolic risk (33, 75), rather than a direct inflammatory effect of low adherence to the MIND diet.

### 4.1 Strengths and limitations

This study has several strengths. First, it includes a comprehensive multidimensional assessment of older adults, encompassing anthropometric and body composition measures, clinical burden, medication, inflammation, lifestyle factors, and patient-centered outcomes (i.e., quality of life, depressive symptoms, and independence in activities of daily living). Second, malnutrition, sarcopenia, sarcopenic obesity and metabolic syndrome were diagnosed according to internationally recognized consensus criteria. Third, the coexistence of malnutrition with both underweight and overweight or obesity was specifically investigated, and the latter was distinguished from sarcopenic obesity phenotypes (76). Fourth, the inclusion of both hospitalized and outpatient older adults enabled comparisons across settings with different levels of disease burden, including an endocrinology outpatient population that remains poorly investigated. Finally, in the absence of GLIM guidance for grading malnutrition severity based on reduced muscle mass (53), study-specific fifth-percentile cut-offs for ASM and calf circumference were calculated and used alongside consensus-based thresholds for other phenotypic criteria.

Some limitations should also be acknowledged. First, the cross-sectional design precludes causal inference. Second, participants were recruited from a single ward and outpatient clinic in Northern Italy and were predominantly Caucasian, which may limit generalizability; however, all eligible patients were consecutively enrolled, which limits selection bias by avoiding discretionary inclusion by investigators. The results should be interpreted within the clinical populations studied and should not be extrapolated to the general community-dwelling older population. Third, the exclusion of patients with severe cognitive impairment, cancer, dysphagia or severe illness may have led to an underestimation of malnutrition and sarcopenia in frailer populations. Fourth, lifestyle and dietary variables were partly self-reported and may be subject to recall bias, although validated questionnaires were used and several variables were objectively measured or retrieved from medical records. BMI relied on demi-span-estimated height, and handgrip strength was measured on the non-dominant hand as the mean of three trials, in accordance with the applied guidelines and manufacturer instructions. These choices should be considered when comparing our findings with studies adopting different protocols. Fifth, gait speed could not be assessed in 27% of participants due to clinical reasons, which may have affected estimates of severe sarcopenia. BIA could not be performed in 62 participants (20.5%), mainly due to clinical contraindications. In most of these cases, calf circumference was used to complete the EWGSOP2 sarcopenia algorithm; however, since current consensus definitions of sarcopenic obesity do not permit this substitution, the diagnosis could not be confirmed in 42 participants with probable sarcopenic obesity, although a subanalysis of the 237 participants with complete data showed a similar prevalence. Biomarker data were also not available for all participants, owing to clinical and logistical factors and to the later incorporation of serum collection for cytokine measurements into the study protocol; this reduced statistical power but was unrelated to participant selection. Sixth, inflammatory, nutritional, and metabolic biomarkers reflect clinical status at the time of assessment. Hospitalization, acute clinical conditions, comorbidities, medications, and other unmeasured factors may influence both nutritional/muscle-related conditions and biomarkers (such as the strength of their association with sarcopenic obesity).

Therefore, the results should be interpreted within the clinical populations studied and should not be extrapolated to the general community-dwelling older population.

## 5 Conclusion

Malnutrition, sarcopenia, sarcopenic obesity, and metabolic syndrome were frequent and overlapping conditions in this cohort of older adults with cardiometabolic diseases, with a greater burden among hospitalized patients. Importantly, excess body weight did not exclude nutritional or muscle impairment, supporting the need for multidimensional assessment beyond BMI alone.

Older age, functional dependence, physical inactivity, and poorer physical health-related quality of life were associated with selected nutritional and muscle-related phenotypes. Among the inflammatory, nutritional, and metabolic biomarkers evaluated, IL-6 showed the most consistent association with age, sarcopenic obesity, and metabolic syndrome, while dietary patterns showed only limited associations with inflammatory and metabolic biomarkers.

These cross-sectional findings support integrated nutritional, functional, body-composition, and cardiometabolic assessment in older adults across clinical settings. Larger multicenter and longitudinal studies are needed to confirm these observations and clarify their temporal and causal relationships.

## Data Availability

The raw data supporting the conclusions of this article will be made available by the authors, without undue reservation.

## Glossary

ADL: Activities of Daily Living
ASM: Appendicular Skeletal Muscle Mass
BIA: Bioelectrical Impedance Analysis
BMI: Body Mass Index
CIRS: Cumulative Illness Rating Scale
DASH: Dietary Approaches to Stop Hypertension
eGFR: estimated Glomerular Filtration Rate
ESPEN/EASO: European Society for Clinical Nutrition and Metabolism/European Association for the Study of Obesity
EVIDENCe-Q: EValuatIon of vitamiN D dEfiCiency Questionnaire
EWGSOP2: European Working Group on Sarcopenia in Older People 2
GDS-15: 15-item Geriatric Depression Scale
GLIM: Global Leadership Initiative on Malnutrition
HDL: High-Density Lipoprotein
ICS: Istituti Clinici Scientifici
IDF: International Diabetes Federation
IL-1β: Interleukin-1 beta
IL-6: Interleukin-6
IL-10: Interleukin-10
IRCCS: Istituto di Ricovero e Cura a Carattere Scientifico
MEDI-LITE: Mediterranean Diet Literature-based Adherence Score
Mets: Metabolic Syndrome
MIND: Mediterranean-DASH Intervention for Neurodegenerative Delay
MMSE: Mini-Mental State Examination
MS: Metabolic Syndrome
MUST: Malnutrition Universal Screening Tool
NCT: National Clinical Trial identifier
OR: Odds Ratio
PASE: Physical Activity Scale for the Elderly
pCO₂: partial pressure of carbon dioxide
PCS-12: Physical Component Summary of the Short Form-12 Health Survey
pO₂: partial pressure of oxygen
PSQI: Pittsburgh Sleep Quality Index
SF-12: 12-item Short Form Health Survey
SIMI-NUTRO: Italian Society of Internal Medicine Nutrition study
SO: Sarcopenic Obesity
SOGLI: Sarcopenic Obesity Global Leadership Initiative
TGF-β: Transforming Growth Factor beta
TNF-α: Tumor Necrosis Factor alpha
WHO: World Health Organization
