# Supplementary material for: Prevalence and clinical characterization of malnutrition, sarcopenia and sarcopenic obesity in older adults with cardiometabolic diseases: SENIOR cross-sectional study
Source: Front Nutr. 2026 Oct 28;13:1927628. doi: 10.3389/fnut.2026.1927628 (PMC13753960; doi:10.3389/fnut.2026.1927628)
Supplement: Supplementary file 1 [file Supplementary_file_1.pdf]

Supplementary Material

Supplementary tables for study methodology

Table S1: Summary of data collection domains, variables and assessment method. Source references for this table are provided below; please note that the numbering differs from the main paper citation sequence.

Domain	Variables	Materials and methods	Cut-offs and notes
Sociodemographic characteristic	Age, sex, ethnicity, educational level, marital status, occupation, housing status, residential area, social support	Structured interviews	/
Clinical history	Comorbidities, medication use, prior hospitalizations	Semi-structured interviews; CIRS (1–3)	CIRS used to assess comorbidity burden
Anthropometry	Height	Validated predictive equations based on knee height and demi-span measurements, suitable for older adults (4,5)<br>-Demi-span: distance from the root of the middle finger to the sternal notch, with the participant's (right) arm raised horizontally to shoulder level and the wrist straight<br>-Knee height: left leg, when possible, was used; participant seated without shoes, knee flexed at a right angle. The tape was extended down the side of the leg, aligned with the lateral malleolus, to the base of the heel	- Demi-span height derived using the Bassey equation, validated for Italian populations (6)<br>- Knee height derived using the Donini equation, developed for the Italian older population (7)
	Body weight	Weighing chair for inpatients and a portable scale for outpatients.	/
	BMI	Body weight / height^2 (kg/m^2)	/
	Waist, mid-upper arm, calf circumferences	Non-elastic tape:<br>- waist: midpoint between the lowest rib and iliac crest (8),<br>- mid-upper arm: midpoint between acromion and olecranon on the non-dominant arm (9),<br>- calf: maximum circumference in the seated position (10)	- Waist: ≤80 cm in women, ≤94 cm in men (11)<br>- Mid-upper arm for ≥65y: >30.5 cm in women, >31 cm in men (12)<br>- Calf: values <31 cm associated with poorer physical performance and increased mortality risk (13)

Supplementary Material

Body composition	Phase angle, fat-free mass, fat mass, appendicular skeletal muscle mass, total body water (including extracellular and intracellular compartments).	BIA (BIA-101, Akern, Florence, Italy), using a low-intensity alternating current (800 μA)	Phase angle (14):<br>- For men: 6.2-6.8 if 59-69y, 4.8-6.4 if 70-80y, 4.5-6.0 if >80 y<br>- For women: 5.4-5.8 if 59-69y, 4.7-5.5 if 70-80y, 5.3-5.6 if >80y.<br>ASM estimated using Sergi equation (15), specifically derived and validated for older European adults[§]
Laboratory biomarkers	Complete blood count,<br>C-reactive protein,<br>Lipid profile (total and HDL cholesterol, triglycerides),<br>Renal and liver function (creatinine, transaminases, gamma-glutamyl transferase, alkaline phosphatase),<br>Muscle enzymes (creatine kinase),<br>Nutritional markers (albumin, prealbumin),<br>Glucose,<br>Insulin,<br>Homocysteine,<br>Vitamins (D, B12, folate),<br>Minerals (sodium, potassium, magnesium, calcium, iron, zinc),<br>Coagulation profile (prothrombin time/INR),<br>Circulating cytokines (IL-1β, IL-6, IL-10, TNF-α, TGF-β).	Fasting serum blood samples (20 ml) were collected for laboratory evaluations, and the remaining serum was stored at -20°C until analysed for circulating cytokines.<br>Specifically, routine laboratory biomarkers (CRP, white blood cell count, platelet count, prealbumin and uric acid) were measured at the institutional clinical laboratory according to standardised procedures routinely adopted for patient care within an accredited quality-controlled setting.<br>Serum IL-6, TNF-α, IL-10 and TGF-β concentrations were measured using commercially available Quantikine ELISA kits (R&D Systems/ Bio-Techne, Minneapolis, MN, USA) according to the manufacturer's instructions*; samples were assayed in duplicate and quality control pools for low, normal, or high concentrations were included	The mean minimum detectable doses for cytokines were: IL-6= 0.368 pg/ml; TNFα= 4.00 pg/ml; IL-1β=1.0 pg/ml; TGFβ= 2.38 pg/ml; IL-10= 3.9 pg/ml
Lifestyle	Smoking status, age at initiation and cessation, smoking intensity (cigarettes/day).	Structured interview	Never, former (no regular smoking in the previous 6 months), or current smokers
	Alcohol drinking status; type, frequency, and average quantity.	Structured interview	Intake expressed as the number of standard units
	Mediterranean Diet and MIND Diet adherence.	MEDI-LITE score (17); MIND diet score (18)	MEDI-LITE cut-off: 8.5;<br>MIND diet score ranges 0-15: higher scores indicate greater adherence to the dietary pattern, associated with a slower rate of cognitive decline
	Physical activity level and activities of daily living.	PASE (19); Barthel Index (20)	PASE <76 considered as insufficient physical activity, in line with Italian population-based references (21)

	Sleep quality.	PSQI questionnaire (22)	PSQI >5 indicates poor sleep quality
	Health-related quality of life.	2 domains of the SF-12 reflecting physical (PCS) and mental (MCS) health (23)	Lower SF-12 scores indicate poorer health status; PCS ≤50 and MCS ≤42 indicate reduced physical and mental health, respectively (24)
	Vitamin D deficiency risk.	EVIDENCe-Q questionnaire (25)	EVIDENCe-Q cut-off score of 23 for severe deficiency, of 21 for deficiency, and of 20 for insufficiency. Participants with EVIDENCe-Q scores <20 had adequate levels of vitamin D
Muscle strength	Non-dominant hand strength.	Handgrip dynamometry (DynEX, Akern srl) (26). Standardised conditions: participant seated with both feet flat on the ground; arm along the side of the body; elbow flexed at 90° with the shoulder in adduction and neutral rotation; wrist in line with the elbow; instrument adjusted to sit comfortably in the hand. Three consecutive maximal-effort trials of 5 seconds each, separated by an adequate recovery interval; the mean of the three trials was used	< 16 kg if women,<br>< 27 kg if men indicate low muscle strength
Physical performance	Gait speed.	4-meter gait speed test with a static start (26)	Gait speed: ≤ 0.8 m/s indicates low physical performance

Abbreviations: CIRS = Cumulative Illness Rating Scale, BMI = Body Mass Index, BIA = Bioelectrical impedance analysis, PASE = Physical Activity Scale for the Elderly, PSQI = Pittsburgh Sleep Quality Index, SF-12 = Short Form-12.

§ Appendicular Skeletal Mass (ASM, kg) was calculated based on the Sergi equation (14):

ASM in women (kg) = −3.964 + (0.227 × Ri) + (0.095 × weight) + (0.064 × Xc)
ASM in men (kg) = −3.964 + (0.227 × Ri) + (0.095 × weight) + 1.384 + (0.064 × Xc)
Resistive index (RI): resistance (R) normalized for height derived by demi-span
Xc: reactance

*Serum concentrations of IL-6, TNF-α, IL-10 and TGF-β1 were measured using commercially available Quantikine ELISA kits (R&D Systems/Bio-Techne, Minneapolis, MN, USA) according to the manufacturer's instructions. Specifically, IL-6 was measured using the Human IL-6 Quantikine ELISA Kit (D6050B; assay range: 3.13–300 pg/mL), TNF-α using the Human TNF-α Quantikine ELISA Kit (DTA00D; assay range: 15.6–1000 pg/mL), IL-10 using the Human IL-10 Quantikine ELISA Kit (D1000B; assay range: 7.8–500 pg/mL), and TGF-β1 using the Human TGF-β1 Quantikine ELISA Kit (DB100C; assay range: 31.2–2000 pg/mL). According to the manufacturer, intra-assay and inter-assay coefficients of variation were <10% for all cytokine assays. All cytokine measurements were performed on serum samples following the manufacturer's recommendations.

1. Miller MD, Paradis CF, Houck PR, Mazumdar S, Stack JA, Rifai AH, et al. Rating chronic medical illness burden in geropsychiatric practice and research: Application of the Cumulative Illness Rating Scale. Psychiatry Res (1992) 41:237–248. doi: 10.1016/0165-1781(92)90005-n

2. Salvi F, Miller MD, Grilli A, Giorgi R, Towers AL, Morichi V, et al. A Manual of Guidelines to Score the Modified Cumulative Illness Rating Scale and Its Validation in Acute Hospitalized Elderly Patients. J Am Geriatr Soc (2008) 56:1926–1931. doi: 10.1111/j.1532-5415.2008.01935.x
3. de Groot V, Beckerman H, Lankhorst GJ, Bouter LM. How to measure comorbidity. a critical review of available methods. Journal of Clinical Epidemiology (2003) 56:221–229. doi: 10.1016/s0895-4356(02)00585-1
4. Weinbrenner T, Vioque J, Barber X, Asensio L. Estimation of Height and Body Mass Index from Demi-Span in Elderly Individuals. Gerontology (2006) 52:275–281. doi: 10.1159/000094608
5. Chumlea WC, Guo SS, Wholihan K, Cockram D, Kuczmarski RJ, Johnson CL. Stature Prediction Equations for Elderly non-Hispanic White, non-Hispanic black, and Mexican-American Persons Developed from NHANES III Data. J Am Diet Assoc (1998) 98:137–142. doi: 10.1016/s0002-8223(98)00036-4
6. Bassey EJ. Demi-span as a measure of skeletal size: an evaluation. Annals of Human Biology. 1986;13(5):499-502.
7. Donini LM, de Felice MR, de Bernardini L, et al. Prediction of stature in the Italian elderly. J Nutr Health Aging. 2000;4(2):72-76.
8. World Health Organization. Waist circumference and waist-hip ratio: report of a WHO expert consultation (2011) https://iris.who.int/handle/10665/44583
9. Eaton–Evans J. Nutritional assessment: anthropometry. In: Caballero B, editor. Encyclopedia of Human Nutrition. Oxford: Elsevier Academic Press (2005). p. 311-318.
10. Norton K. Standards for anthropometry assessment. In: Norton K, Eston R, editors. Kinanthropometry and exercise physiology. 4th ed. London: Routledge (2018). p. 68-137.
11. Alberti KGMM, Zimmet P, Shaw J. Metabolic syndrome—a new world-wide definition. A Consensus Statement from the International Diabetes Federation. Diabet Med (2006) 23:469–480. doi: 10.1111/j.1464-5491.2006.01858.x
12. Bedogni G, Borghi A, Battistini NC. Manuale di valutazione antropometrica dello stato nutrizionale. Milano: Edra (2001). 172 p
13. Landi F, Onder G, Russo A, Liperoti R, Tosato M, Martone AM, et al. Calf circumference, frailty and physical performance among older adults living in the community. Clin Nutr (2014) 33:539–544. doi: 10.1016/j.clnu.2013.07.013
14. Mattiello R, Amaral MA, Mundstock E, Ziegelmann PK. Reference values for the phase angle of the electrical bioimpedance: Systematic review and meta-analysis involving more than 250,000 subjects. Clin Nutr (2020) 39:1411–1417. doi: 10.1016/j.clnu.2019.07.004
15. Sergi G, De Rui M, Veronese N, et al. Assessing appendicular skeletal muscle mass with bioelectrical impedance analysis in free-living Caucasian older adults. Clin Nutr. 2015;34(4):667-673. doi:10.1016/j.clnu.2014.07.010
16. EpiCentro - Istituto Superiore di Sanità (ISS). Sorveglianza PASSI: Consumo di alcol (2026). https://www.epicentro.iss.it/passi/indicatori/alcol
17. Sofi F, Dinu M, Pagliai G, Marcucci R, Casini A. Validation of a literature-based adherence score to Mediterranean diet: the MEDI-LITE score. Int J Food Sci Nutr (2017) 68:757–762. doi: 10.1080/09637486.2017.1287884
18. Morris MC, Tangney CC, Wang Y, Sacks FM, Barnes LL, Bennett DA, et al. MIND diet slows cognitive decline with aging. Alzheimers Dement (2015) 11:1015–1022. doi: 10.1016/j.jalz.2015.04.011
19. Covotta A, Gagliardi M, Berardi A, Maggi G, Pierelli F, Mollica R, et al. Physical Activity Scale for the Elderly: Translation, Cultural Adaptation, and Validation of the Italian Version. Curr Gerontol Geriatr Res (2018) 2018:1–7. doi: 10.1155/2018/8294568
20. Galeoto G, Lauta A, Palumbo A, Castiglia SF, Mollica R, Santilli V, et al. The Barthel Index: Italian translation, adaptation and validation. Int J Neurol Neurother. (2015) 2:1-7.
21. EpiCentro - Istituto Superiore di Sanità (ISS). Sorveglianza PASSI: Attività fisica secondo le raccomandazioni dell'OMS (2025) https://www.epicentro.iss.it/passi/dati/attivita-oms
22. Curcio G, Tempesta D, Scarlata S, Marzano C, Moroni F, Rossini PM, et al. Validity of the Italian Version of the Pittsburgh Sleep Quality Index (PSQI). Neurol Sci (2012) 34:511–519. doi: 10.1007/s10072-012-1085-y
23. Kodraliu P, Mosconi N, Groth GG. Subjective health status assessment: evaluation of the Italian version of the SF-12 Health Survey. Results from the MiOS Project. J Epidemiol Biostat (2001) 6:305–316. doi: 10.1080/135952201317080715
24. Ware J, Kosinski M, Keller S. SF-12: How to score the SF-12 Physical and Mental Summary Scales. Boston, MA: The Health Institute, New England Medical Center (1995). 130 p
25. De Giuseppe R, Tomasinelli CE, Cena H, Braschi V, Giampieri F, Preatoni G, et al. Development of a Short Questionnaire for the Screening for Vitamin D Deficiency in Italian Adults: The EVIDENCe-Q Project. Nutrients (2022) 14:1772. doi: 10.3390/nu14091772

26. Cruz-Jentoft AJ, Bahat G, Bauer J, Boirie Y, Bruyère O, Cederholm T, et al. Sarcopenia: revised European consensus on definition and diagnosis. Age Ageing (2018) 48:16–31. doi: 10.1093/ageing/afy169.

Supplementary Material

Table S2: Malnutrition diagnosis and severity criteria adopted in the current study. Reference: Cederholm T, Jensen GL, Correia MITD, Gonzalez MC, Fukushima R, Higashiguchi T, et al. GLIM criteria for the diagnosis of malnutrition – A consensus report from the global clinical nutrition community. Clin Nutr (2019) 38:1–9. doi: 10.1016/j.clnu.2018.08.002.

<table>
<tr><td colspan="2">Malnutrition diagnosis</td></tr>
<tr><td>Phenotypic criteria</td><td>Etiologic criteria</td></tr>
<tr><td>● Unintentional weight loss:
>5% within past 6 months,
or >10% beyond 6 months.
● Low BMI (kg/m^2):
<20 if <70 years of age,
or <22 if >70 years of age.
● Reduced muscle mass:
ASM <15 kg if women, ASM <20 kg if men;
or CC <31 cm for both sexes.</td><td>● Reduced food intake or assimilation
● Inflammatory acute or chronic conditions.</td></tr>
<tr><td colspan="2">Malnutrition severity: Phenotypic criteria</td></tr>
<tr><td>Moderate malnutrition</td><td>Severe malnutrition</td></tr>
<tr><td>● Unintentional weight loss:
5-10% within past 6 months,
or >10-20% beyond 6 months.
● Low BMI (kg/m^2):
<20 if <70 years of age,
or <22 if ≥70 years of age.
● Mild to moderate reduction of muscle mass:
ASM <15 kg if women, ASM <20 kg if men;
or CC <31 cm for both sexes.</td><td>● Unintentional weight loss:
>10% within past 6 months,
or >20% beyond 6 months
● Low BMI (kg/m2):
<18.5 if <70 years of age,
or <20 if ≥70 years of age
● Severe reduction of muscle mass:
ASM or CC < 5 pct (*)</td></tr>
</table>

(*) Percentile values of ASM (male: p5 = 16.469 kg; female: p5 = 11.361 kg); percentile value of CC (for both sexes p5 = 27 cm)

Abbreviations: BMI = BMI = Body Mass Index, ASM = appendicular skeletal muscle mass, CC = calf circumference.

Phenotypic criteria included unintentional weight loss within the previous 6-12 months, low BMI, and reduced muscle mass assessed through bioelectrical impedance analysis (ASM, kg), or through calf circumference when BIA was not feasible. Etiologic criteria included reduced food intake or assimilation, as assessed by clinical history, and/or the presence of disease burden or inflammatory conditions. For malnutrition severity, sex-specific distributions of ASM and calf circumference were calculated, and thresholds based on the 5th percentiles were applied. Values below the 5th percentile were classified as severe (stage II) malnutrition.

Malnutrition risk screening (MUST) and malnutrition diagnosis (GLIM criteria) were applied independently in all participants. According to the recent GLINTS consensus, validated malnutrition screening tools can be used if the malnutrition risk is unknown. Nonetheless, the GLIM criteria may be applied directly, without prior screening, if a high risk of malnutrition is already evident (Klek et al, 2026). Since SENIOR participants were aged >= 65 years, mainly hospitalized, multimorbid and receiving polypharmacy, and therefore represented a population at high risk of malnutrition, the GLIM criteria were applied to all participants regardless of the MUST result. Accordingly, MUST was used to estimate the prevalence of malnutrition risk, whereas the GLIM criteria were used to diagnose malnutrition.

Supplementary Material

Table S3. International standardized diagnostic criteria and cut-offs adopted in the current study. Source references for this table are provided below; please note that the numbering differs from the main paper citation sequence.

<table>
<tr><td colspan="3">Sarcopenia (EWGSOP 2) (1)</td></tr>
<tr><td>Probable sarcopenia:
low muscle strength.
Handgrip:
< 16 kg for women,
< 27 kg for men</td><td>Confirmed diagnosis:
low muscle quantity.
ASM by BIA:
< 15 kg for women,
< 20 kg for men.
CC (if BIA was not available):
<31 cm.</td><td>Severe sarcopenia:
low physical performance.
Gait speed: ≤ 0.8 m/s</td></tr>
<tr><td colspan="3">Sarcopenic obesity (ESPEN/EASO Consensus) (2-4)</td></tr>
<tr><td>Screening criteria.
Both central obesity and sarcopenia indicators were met:
A) elevated waist circumference (≥80 cm in women, ≥94 cm in men) or a BMI ≥ 30 kg/m^2;
B) indicators of sarcopenia (e.g., clinical symptoms or suspicion risk factors).</td><td>Diagnostic criteria.
Sequential assessment of:
1. altered muscle strength: handgrip <16 kg for women, <27 kg for men;
2. altered body composition: FM% >43% for women, >31% for men
<u>and</u>
ASM/W*100 <25.72% for men, <19.43 % for women</td><td>Staging criteria.
Stage I: absence of clinical complications;
Stage II: at least one of the following complications: endocrine-metabolic diseases (including hepatic steatosis), cardiovascular diseases, respiratory diseases, neurodegenerative diseases, and functional disability (assessed through Barthel index).</td></tr>
<tr><td colspan="3">Metabolic syndrome (IDF Consensus diagnosis criteria) (5)</td></tr>
<tr><td>Mandatory criteria: Central obesity:
≥80 cm in women, or ≥94 cm in men,
or a BMI ≥ 30 kg/m^2.</td><td colspan="2">In addition, presence of at least 2 of the following criteria:
i) elevated triglycerides (≥150 mg/dL) or ongoing pharmacological treatment for hypertriglyceridemia;
ii) reduced HDL cholesterol (<40 mg/dL in men and <50 mg/dL in women) or specific treatment for this lipid abnormality;
iii) current antihypertensive treatment after hypertension diagnosis;
iv) elevated fasting plasma glucose (≥100 mg/dL) or previously diagnosed type 2 diabetes.</td></tr>
</table>

Abbreviations: ASM = appendicular skeletal muscle mass, BIA = Bioelectrical impedance analysis, CC = calf circumference, BMI = Body Mass Index, HDL = high-density lipoprotein

1. Cruz-Jentoft AJ, Bahat G, Bauer J, Boirie Y, Bruyère O, Cederholm T, et al. Sarcopenia: revised European consensus on definition and diagnosis. Age Ageing (2018) 48:16–31. doi: 10.1093/ageing/afy169

2. Donini LM, Busetto L, Bischoff SC, Cederholm T, Ballesteros-Pomar MD, Batsis JA, et al. Definition and Diagnostic Criteria for Sarcopenic Obesity: ESPEN and EASO Consensus Statement. Obes Facts (2022) 15:321–35. doi: 10.1159/000521241
3. Marini E, Sulis S, Vorobel'ová L, Stagi S. Specific bioelectrical vectors pattern in individuals with sarcopenic obesity. Clin Nutr(2024) 43:620–628. doi: 10.1016/j.clnu.2024.01.024
4. Levine ME, Crimmins EM. The Impact of Insulin Resistance and Inflammation on the Association Between Sarcopenic Obesity and Physical Functioning. Obesity (2012) 20:2101–2106. doi: 10.1038/oby.2012.20
5. Alberti KGMM, Zimmet P, Shaw J. Metabolic syndrome—a new world-wide definition. A Consensus Statement from the International Diabetes Federation. Diabet Med (2006) 23:469–480. doi: 10.1111/j.1464-5491.2006.01858.x

Supplementary Material

Supplementary Figure 1: Flowchart of participant recruitment and enrollment. It shows separately the assessment of patients in the Internal Medicine ward and in the Endocrinology outpatient clinic. The diagram reports the number of older adults screened, excluded due to ineligibility, declining participation, or subsequently excluded for other reasons, as well as the final number of patients enrolled in the study and included in the analyses. Note: "Absence" refers to patients who could not be assessed because they had been discharged or were temporarily unavailable while undergoing diagnostic or medical procedures.

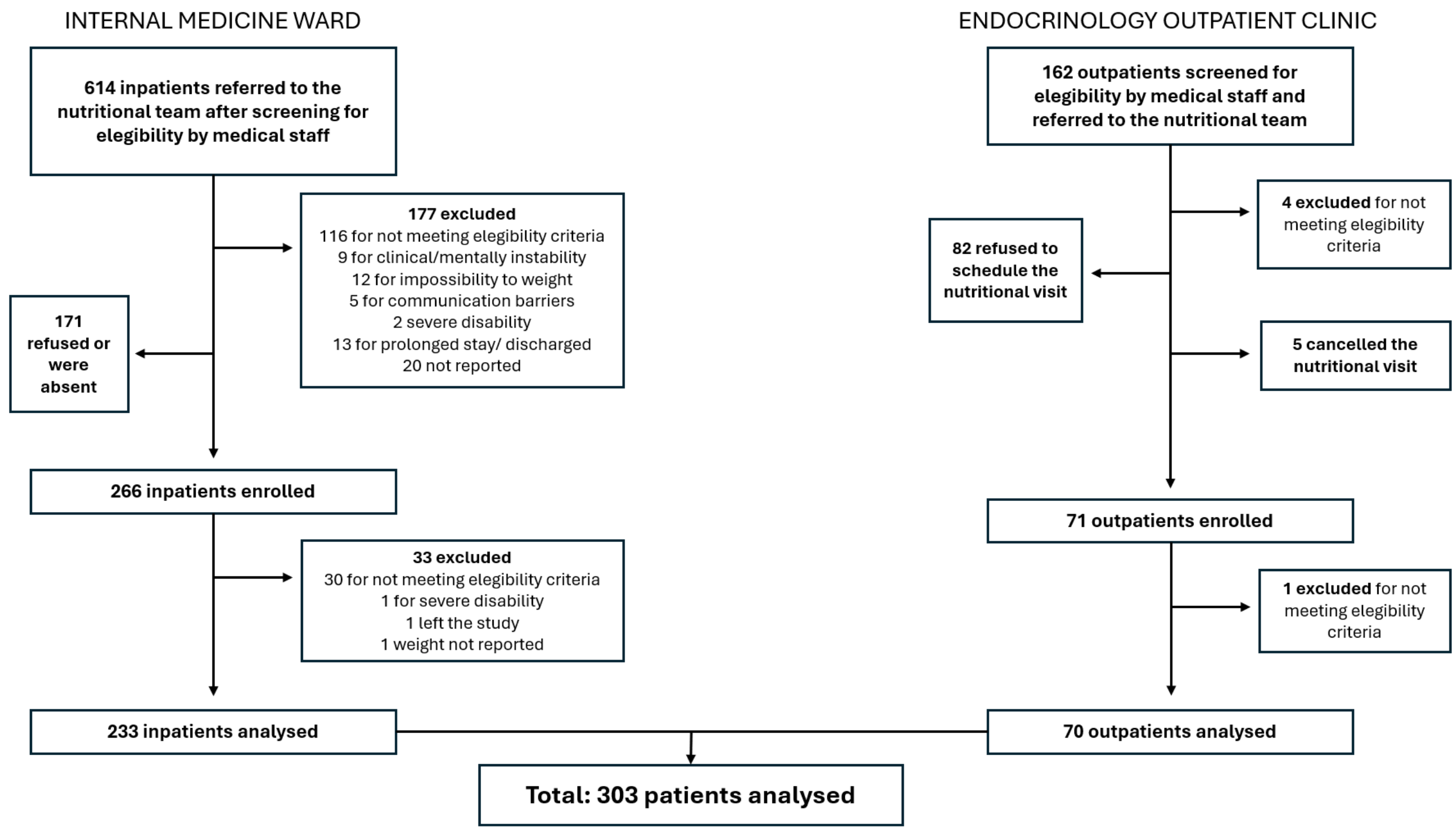

List of available data and missing data for each variable, with related reasons

Sociodemographic variables

- Sex: 303
- Age: 303
- Education level: 301 (2 missing data due to data collection error)
- Civil status: 303
- Housing status: 301 (2 missing data: 1 for data collection error, 1 due to the patient's refusal to respond)
- Living area: 299 (4 missing data due to data collection error)
- Social support: 301 (2 missing data due to data collection error)
- Current occupation: 303

Nutritional assessment

- Body Mass Index, BMI: 303
- Body weight: 303
- Weight loss in the past 3-6 months: 303
- Height derived from demi-span: 303
- Height derived from knee height: 299 (4 missing data: 3 patients could not bend the knee, 1 patient refused for pain and fatigue)
- Waist circumference: 288 (15 missing data, primarily due to severe mobility limitations preventing the required assessment. Specifically, 14 participants were bedridden or unable to assume the required position because of impaired mobility, physical restraints, vertigo, or pain. For 1 outpatient no reason was documented)
- Mid-arm circumference: 260 (43 missing data: for the first 36 participants it was not collected due to the study protocol, since it was later added through an amendment; 1 participant was bedridden in conditions limiting the possibility of properly collecting it. For 1 inpatient and for 5 outpatients no reason was documented)
- Calf circumference: 259 (44 missing data: for the first 36 participants it was not collected due to the study protocol, since it was later added through an amendment; 1 participant was bedridden with prosthesis. For 7 participants no reason was documented)
- Appendicular skeletal mass: 241 (62 missing data: for 42, it was not collected due to contraindication for accurate data acquisition such as prostheses or metallic implants, or patient safety concerns such as pacemakers, implantable cardioverter-defibrillators or other electrical stimulation devices; 1 participant was unable to lie; 1 refused to remove the graduated compression stockings. For 18 participants no reason was documented)
- Appendicular skeletal mass index: 241 (62 missing data) → same as above
- Fat-free mass: 241 (62 missing data) → same as above
- Fat mass: 241 (62 missing data) → same as above
- Phase angle: 241 (62 missing data) → same as above

Clinical variables

- Cumulative Illness Rating Scale: 302 (1 missing data for lack of access to medical documentation)
- Polypharmacy: 301
- Prior hospitalizations in the last year: 303

Supplementary Material

- Length of hospital stay: 303

Lifestyle

- Adherence to Mediterranean diet (MEDI-LITE score): 301 (2 missing data due to routine medical workflow constraints, i.e. medical examinations and diagnostic tests to be performed)
- Adherence to MIND diet (MIND diet score): 301 (2 missing data due to routine medical workflow constraints, i.e. medical examinations and diagnostic tests to be performed)
- Alcohol consumption (structured interview): 303
- Smoking habits (structured interview): 303
- Physical activity level (PASE score): 301 (2 missing data due to routine medical workflow constraints, i.e. medical examinations and diagnostic tests to be performed)
- Activities of daily living (Barthel Index): 301 (2 missing data due to routine medical workflow constraints, i.e. medical examinations and diagnostic tests to be performed)
- Sleep quality (PSQI score): 301 (2 missing data due to routine medical workflow constraints, i.e. medical examinations and diagnostic tests to be performed)
- Quality of life (SF-12): 299 (4 missing data due to routine medical workflow constraints, i.e. medical examinations and diagnostic tests to be performed)
- Depression (GDS-15): 294 (9 missing data: 3 missing data due to routine medical workflow constraints, i.e. medical examinations and diagnostic tests to be performed; 5 participants refuse to reply to one or more questions; 1 participant started to cry, thus the filling was interrupted)

Functional assessment

- Strength (handgrip): 298 (5 missing data: 1 participant was bedridden under physical restraints; 1 suffered from hypokinetic-rigid Parkinson's disease; 1 from tetraparesis; 1 from myasthenia gravis. For 1 outpatient no reason was documented)
- Locomotion (4-m gait speed): 219 (84 missing data: 81 participants were unable to perform the test due to patient safety risk due to clinical instability and/or high risk of falls, due to dizziness or vertigo, due to pain while walking related to underlying clinical conditions, due to bedridden status or requiring wheelchair transport, or due to medical devices such as intravenous infusions, oxygen therapy, and urinary catheter. For 5 participants the reason was not documented)

Diagnosis

- Malnutrition risk (MUST questionnaire): 301 (2 missing data: 1 due to lack of information on reduced food intake in the past 5 days; 1 due to lack of information on acute diseases)
- Malnutrition diagnosis (GLIM): 303
- Sarcopenia diagnosis (EWGSOP2): 298 (5 missing data due to the lack of handgrip strength)
- Probable sarcopenic obesity (ESPEN/EASO): 298 (5 missing data due to the lack of handgrip strength)
- Sarcopenic obesity (ESPEN/EASO): 237 (67 missing data, 5 due to missing handgrip strength and 62 due to lack of BIA assessments. Since one participant lacked both handgrip and BIA, these are 67 missing measurements but 66 unique participants lacking complete data)
- Metabolic syndrome (IDF criteria): 303
- Cardiovascular diseases: 302 (1 missing data for lack of access to medical documentation)
- Endocrine-metabolic diseases: 302 (1 missing data for lack of access to medical documentation)

Inflammation

Missing laboratory data were attributable to clinical and logistical factors commonly encountered in hospitalized older adults, including difficult venous access, patient refusal (in a minority of cases), insufficient sample volume, pre-analytical issues, or discharge before blood sampling. Missingness therefore reflected real-world constraints of data collection rather than selective exclusion of participants. Furthermore, recruitment started before serum collection for cytokine measurements had been incorporated into the study protocol.

- C-reactive protein, CRP: 198 (105 missing data)
- White blood cell count: 300 (3 missing data)
- Platelet count: 298 (5 missing data)
- Prealbumin: 165 (138 missing data)
- Uric acid: 151 (152 missing data)
- Interleukin-6 (IL-6): 212 (91 missing data)
- Tumor necrosis factor-α (TNF-α): 211 (92 missing data)
- Transforming growth factor-β (TGF-β): 201 (102 missing data)
- Interleukin-1 β (IL-1β): 194 (109 missing data)
- Interleukin-10 (IL-10): 195 (108 missing data)

Supplementary Material

Literature supporting Figure 1. The aim was to delineate the links between malnutrition (GLIM), sarcopenia (EWGSOP2), sarcopenic obesity (ESPEN/EASO), cardiometabolic diseases, and metabolic syndrome. Please note that the numbering differs from the main paper citation sequence.

1. Albukhari S, Abulmeaty MMA, Alguwaihes AM, Shoqeair M, Aldisi D, Alhamdan A. GLIM Criteria for Assessment of Malnutrition in Saudi Patients with Type 2 Diabetes. Nutrients. 2023;15(4):897. Published 2023 Feb 10. doi:10.3390/nu15040897
2. Atkins JL, Whincup PH, Morris RW, Lennon LT, Papacosta O, Wannamethee SG. Sarcopenic obesity and risk of cardiovascular disease and mortality: a population-based cohort study of older men. J Am Geriatr Soc. 2014;62(2):253-260. doi:10.1111/jgs.12652
3. Bano G, Trevisan C, Carraro S, et al. Inflammation and sarcopenia: A systematic review and meta-analysis. Maturitas. 2017;96:10-15. doi:10.1016/j.maturitas.2016.11.006
4. Barazzoni R, Sievenpiper JL, Genton L, et al. Sarcopenic diabetes is an under-recognized and unmet clinical priority. A call for action from the European Society for Clinical Nutrition and Metabolism and the Diabetes Nutrition Study Group. Clin Nutr. 2025;55:208-218. doi:10.1016/j.clnu.2025.10.007
5. Beals JW, Burd NA, Moore DR, van Vliet S. Obesity Alters the Muscle Protein Synthetic Response to Nutrition and Exercise. Front Nutr. 2019;6:87. Published 2019 Jun 13. doi:10.3389/fnut.2019.00087
6. Beals JW, Skinner SK, McKenna CF, et al. Altered anabolic signalling and reduced stimulation of myofibrillar protein synthesis after feeding and resistance exercise in people with obesity. J Physiol. 2018;596(21):5119-5133. doi:10.1113/JP276210
7. Beaudart C, Sanchez-Rodriguez D, Locquet M, Reginster JY, Lengelé L, Bruyère O. Malnutrition as a Strong Predictor of the Onset of Sarcopenia. Nutrients. 2019;11(12):2883. Published 2019 Nov 27. doi:10.3390/nu11122883
8. Benz E, Pinel A, Guillet C, et al. Sarcopenia and Sarcopenic Obesity and Mortality Among Older People. JAMA Netw Open. 2024;7(3):e243604. Published 2024 Mar 4. doi:10.1001/jamanetworkopen.2024.3604
9. Bian AL, Hu HY, Rong YD, Wang J, Wang JX, Zhou XZ. A study on relationship between elderly sarcopenia and inflammatory factors IL-6 and TNF-α. Eur J Med Res. 2017;22(1):25. doi:10.1186/s40001-017-0266-9
10. Breen L, Phillips SM. Skeletal muscle protein metabolism in the elderly: Interventions to counteract the 'anabolic resistance' of ageing. Nutr Metab (Lond). 2011;8:68. Published 2011 Oct 5. doi:10.1186/1743-7075-8-68
11. Cederholm T, Jensen GL, Ballesteros-Pomar MD, et al. Guidance for assessment of the inflammation etiologic criterion for the GLIM diagnosis of malnutrition: A modified Delphi approach. Clin Nutr. 2024;43(5):1025-1032. doi:10.1016/j.clnu.2023.11.026
12. Cederholm T, Jensen GL, Correia MITD, et al. GLIM criteria for the diagnosis of malnutrition - A consensus report from the global clinical nutrition community. Clin Nutr. 2019;38(1):1-9. doi:10.1016/j.clnu.2018.08.002
13. Chandrashekhar Iyer L, Vaishali K, Babu AS. Prevalence of sarcopenia in heart failure: A systematic review. Indian Heart J. 2023;75(1):36-42. doi:10.1016/j.ihj.2022.12.004
14. Coelho-Junior HJ, Calvani R, Azzolino D, et al. Protein Intake and Sarcopenia in Older Adults: A Systematic Review and Meta-Analysis. Int J Environ Res Public Health. 2022;19(14):8718. Published 2022 Jul 18. doi:10.3390/ijerph19148718
15. Coelho-Júnior HJ, Calvani R, Picca A, et al. Biomarkers Linked to Malnutrition Identified According to GLIM Criteria Among Older Community-Dwelling Adults: Results from the ilSIRENTE Study. Nutrients. 2025;17(22):3543. Published 2025 Nov 13. doi:10.3390/nu17223543
16. Cruz-Jentoft AJ, Bahat G, Bauer J, et al. Sarcopenia: revised European consensus on definition and diagnosis. Age Ageing. 2019;48(1):16-31. doi:10.1093/ageing/afy169
17. Damluji AA, Alfaraidhy M, AlHajri N, et al. Sarcopenia and Cardiovascular Diseases. Circulation. 2023;147(20):1534-1553. doi:10.1161/CIRCULATIONAHA.123.064071
18. de Freitas MM, de Oliveira VLP, Grassi T, et al. Difference in sarcopenia prevalence and associated factors according to 2010 and 2018 European consensus (EWGSOP) in elderly patients with type 2 diabetes mellitus. Exp Gerontol. 2020;132:110835. doi:10.1016/j.exger.2020.110835
19. Demirdağ F, Kolbaşı EN, Yildiz Guler K. The association between sarcopenic obesity and malnutrition in community-dwelling older adults. Age Ageing. 2025;54(2):afaf040. doi:10.1093/ageing/afaf040
20. Deutz NE, Bauer JM, Barazzoni R, et al. Protein intake and exercise for optimal muscle function with aging: recommendations from the ESPEN Expert Group. Clin Nutr. 2014;33(6):929-936. doi:10.1016/j.clnu.2014.04.007
21. Donini LM, Busetto L, Bischoff SC, et al. Definition and Diagnostic Criteria for Sarcopenic Obesity: ESPEN and EASO Consensus Statement. Obes Facts. 2022;15(3):321-335. doi:10.1159/000521241

22. Duan R, Chen S, Li S, et al. Association between GLIM diagnosed malnutrition and 18-month mortality in hospitalized adults with congestive heart failure: A prospective cohort study. JPEN J Parenter Enteral Nutr. 2025;49(5):633-642. doi:10.1002/jpen.2760
23. Farmer RE, Mathur R, Schmidt AF, et al. Associations Between Measures of Sarcopenic Obesity and Risk of Cardiovascular Disease and Mortality: A Cohort Study and Mendelian Randomization Analysis Using the UK Biobank. J Am Heart Assoc. 2019;8(13):e011638. doi:10.1161/JAHA.118.011638
24. Feng Z, Xia J, Yu J, et al. Pathophysiological Mechanisms Underlying Sarcopenia and Sarcopenic Obesity: A Systematic Review and Meta-Analysis of Biomarker Evidence. Int J Mol Sci. 2025;26(11):5113. Published 2025 May 26. doi:10.3390/ijms26115113
25. Frisoli Junior A, Diniz AR, Duque G, et al. Sarcopenia predicts mortality in older adults with cardiovascular disease: concordant findings from SDOC and EWGSOP II criteria despite limited role of low lean mass. Maturitas. 2025;199:108618. doi:10.1016/j.maturitas.2025.108618
26. Gortan Cappellari G, Guillet C, Poggiogalle E, et al. Sarcopenic obesity research perspectives outlined by the sarcopenic obesity global leadership initiative (SOGLI) - Proceedings from the SOGLI consortium meeting in rome November 2022. Clin Nutr. 2023;42(5):687-699. doi:10.1016/j.clnu.2023.02.018
27. Gortan Cappellari G, Semolic A, Zanetti M, et al. Sarcopenic obesity in free-living older adults detected by the ESPEN-EASO consensus diagnostic algorithm: Validation in an Italian cohort and predictive value of insulin resistance and altered plasma ghrelin profile. Metabolism. 2023;145:155595. doi:10.1016/j.metabol.2023.155595
28. Gortan Cappellari G, Semolic A, Zanetti M, et al. Sarcopenic obesity in free-living older adults detected by the ESPEN-EASO consensus diagnostic algorithm: Validation in an Italian cohort and predictive value of insulin resistance and altered plasma ghrelin profile. Metabolism. 2023;145:155595. doi:10.1016/j.metabol.2023.155595
29. Guillet C, Boirie Y. Insulin resistance: a contributing factor to age-related muscle mass loss?. Diabetes Metab. 2005;31 Spec No 2:5S20-5S26. doi:10.1016/s1262-3636(05)73648-x
30. Guillet C, Delcourt I, Rance M, et al. Changes in basal and insulin and amino acid response of whole body and skeletal muscle proteins in obese men. J Clin Endocrinol Metab. 2009;94(8):3044-3050. doi:10.1210/jc.2008-2216
31. Haran PH, Rivas DA, Fielding RA. Role and potential mechanisms of anabolic resistance in sarcopenia. J Cachexia Sarcopenia Muscle. 2012;3(3):157-162. doi:10.1007/s13539-012-0068-4
32. Hirose S, Matsue Y, Kamiya K, et al. Prevalence and prognostic implications of malnutrition as defined by GLIM criteria in elderly patients with heart failure. Clin Nutr. 2021;40(6):4334-4340. doi:10.1016/j.clnu.2021.01.014
33. Huang W, Deng S, Liu S, et al. Association of metabolic syndrome and sarcopenia with all-cause and cardiovascular mortality: a prospective cohort study based on the NHANES. Front Endocrinol (Lausanne). 2024;15:1346669. Published 2024 Mar 26. doi:10.3389/fendo.2024.1346669
34. Jauffret C, Périchon R, Lamer A, Cortet B, Chazard E, Paccou J. Association between sarcopenia and risk of major adverse cardiac and cerebrovascular events-UK Biobank database. J Am Geriatr Soc. 2024;72(3):693-706. doi:10.1111/jgs.18664
35. Jiang M, Ren X, Han L, Zheng X. Associations between sarcopenic obesity and risk of cardiovascular disease: A population-based cohort study among middle-aged and older adults using the CHARLS. Clin Nutr. 2024;43(3):796-802. doi:10.1016/j.clnu.2024.02.002
36. Joaquín C, Alonso N, Lupón J, et al. Nutritional Status According to the GLIM Criteria in Patients with Chronic Heart Failure: Association with Prognosis. Nutrients. 2022;14(11):2244. Published 2022 May 27. doi:10.3390/nu14112244
37. Kalinkovich A, Livshits G. Sarcopenic obesity or obese sarcopenia: A cross talk between age-associated adipose tissue and skeletal muscle inflammation as a main mechanism of the pathogenesis. Ageing Res Rev. 2017;35:200-221. doi:10.1016/j.arr.2016.09.008
38. Kayhan Kocak FO, Altın Z, Kızıltaş A. Malnutrition by GLIM and its overlap with sarcopenic obesity in older adults with elevated body mass index: A retrospective cross-sectional study. Nutr Clin Pract. 2026;41(3):799-810. doi:10.1002/ncp.70086.
39. Khadra D, Itani L, Chebaro Y, et al. Association Between Sarcopenic Obesity and Metabolic Syndrome in Adults: A Systematic Review and Meta-Analysis. Curr Cardiol Rev. 2020;16(2):153-162. doi:10.2174/1573403X16666200214104122
40. Khadra D, Itani L, Tannir H, Kreidieh D, El Masri D, El Ghoch M. Association between sarcopenic obesity and higher risk of type 2 diabetes in adults: A systematic review and meta-analysis. World J Diabetes. 2019;10(5):311-323. doi:10.4239/wjd.v10.i5.311
41. Komici K, Dello Iacono A, De Luca A, et al. Adiponectin and Sarcopenia: A Systematic Review With Meta-Analysis. Front Endocrinol (Lausanne). 2021;12:576619. Published 2021 Apr 15. doi:10.3389/fendo.2021.576619
42. Kootaka Y, Kamiya K, Hamazaki N, et al. The GLIM criteria for defining malnutrition can predict physical function and prognosis in patients with cardiovascular disease. Clin Nutr. 2021;40(1):146-152. doi:10.1016/j.clnu.2020.04.038

43. Kwon SS, Lee SG, Lee YH, Lim JB, Kim JH. Homeostasis model assessment of insulin resistance in a general adult population in Korea: additive association of sarcopenia and obesity with insulin resistance. Clin Endocrinol (Oxf). 2017;86(1):44-51. doi:10.1111/cen.13233
44. Lengelé L, Bruyère O, Beaudart C, Reginster JY, Locquet M. Malnutrition, assessed by the Global Leadership Initiative on Malnutrition (GLIM) criteria but not by the mini nutritional assessment (MNA), predicts the incidence of sarcopenia over a 5-year in the SarcoPhAge cohort. Aging Clin Exp Res. 2021;33(6):1507-1517. doi:10.1007/s40520-021-01880-5
45. Levine ME, Crimmins EM. The impact of insulin resistance and inflammation on the association between sarcopenic obesity and physical functioning. Obesity (Silver Spring). 2012;20(10):2101-2106. doi:10.1038/oby.2012.20
46. Lu W, Feng W, Lai J, Yuan D, Xiao W, Li Y. Role of adipokines in sarcopenia. Chin Med J (Engl). 2023;136(15):1794-1804. Published 2023 Aug 5. doi:10.1097/CM9.0000000000002255
47. Lynch GM, Murphy CH, Castro EM, Roche HM. Inflammation and metabolism: the role of adiposity in sarcopenic obesity. Proc Nutr Soc. Published online July 16, 2020. doi:10.1017/S0029665120007119
48. Minnetti M, Pierantozzi G, Aubertin-Leheudre M, et al. Current application and future directions for the sarcopenic obesity Global Leadership Initiative (SOGLI) diagnostic algorithm. Clin Nutr. Published online April 28, 2026. doi:10.1016/j.clnu.2026.106675
49. Moore DR. Keeping older muscle "young" through dietary protein and physical activity. Adv Nutr. 2014;5(5):599S-607S. doi:10.3945/an.113.005405
50. Murton AJ, Marimuthu K, Mallinson JE, et al. Obesity Appears to Be Associated With Altered Muscle Protein Synthetic and Breakdown Responses to Increased Nutrient Delivery in Older Men, but Not Reduced Muscle Mass or Contractile Function. Diabetes. 2015;64(9):3160-3171. doi:10.2337/db15-0021
51. Park MJ, Choi KM. Interplay of skeletal muscle and adipose tissue: sarcopenic obesity. Metabolism. 2023;144:155577. doi:10.1016/j.metabol.2023.155577
52. Paulussen KJM, McKenna CF, Beals JW, Wilund KR, Salvador AF, Burd NA. Anabolic Resistance of Muscle Protein Turnover Comes in Various Shapes and Sizes. Front Nutr. 2021;8:615849. Published 2021 May 5. doi:10.3389/fnut.2021.615849
53. Pourhassan M, Cederholm T, Trampisch U, Volkert D, Wirth R. Inflammation as a diagnostic criterion in the GLIM definition of malnutrition-what CRP-threshold relates to reduced food intake in older patients with acute disease?. Eur J Clin Nutr. 2022;76(3):397-400. doi:10.1038/s41430-021-00977-4
54. Prado CM, Batsis JA, Donini LM, Gonzalez MC, Siervo M. Sarcopenic obesity in older adults: a clinical overview. Nat Rev Endocrinol. 2024;20(5):261-277. doi:10.1038/s41574-023-00943-z
55. Remelli F, Maietti E, Abete P, et al. Prevalence of obesity and diabetes in older people with sarcopenia defined according to EWGSOP2 and FNHI criteria. Aging Clin Exp Res. 2022;34(1):113-120. doi:10.1007/s40520-021-01949-1
56. Sanz-París A, Martín-Palmero A, Gomez-Candela C, et al. GLIM Criteria at Hospital Admission Predict 8-Year All-Cause Mortality in Elderly Patients With Type 2 Diabetes Mellitus: Results From VIDA Study. JPEN J Parenter Enteral Nutr. 2020;44(8):1492-1500. doi:10.1002/jpen.1781
57. Schaap LA, Pluijm SM, Deeg DJ, Visser M. Inflammatory markers and loss of muscle mass (sarcopenia) and strength. Am J Med. 2006;119(6):526.e9-526.e5.26E17. doi:10.1016/j.amjmed.2005.10.049
58. Scott D, Blyth F, Naganathan V, et al. Sarcopenia prevalence and functional outcomes in older men with obesity: Comparing the use of the EWGSOP2 sarcopenia versus ESPEN-EASO sarcopenic obesity consensus definitions. Clin Nutr. 2023;42(9):1610-1618. doi:10.1016/j.clnu.2023.07.014
59. Seko T, Akasaka H, Koyama M, et al. Preserved Lower Limb Muscle Mass Prevents Insulin Resistance Development in Nondiabetic Older Adults. J Am Med Dir Assoc. 2023;24(3):376-381.e1. doi:10.1016/j.jamda.2022.12.002
60. Sobestiansky S, Åberg AC, Cederholm T. Sarcopenia and malnutrition in relation to mortality in hospitalised patients in geriatric care - predictive validity of updated diagnoses. Clin Nutr ESPEN. 2021;45:442-448. doi:10.1016/j.clnesp.2021.07.002
61. Tardif N, Salles J, Guillet C, et al. Muscle ectopic fat deposition contributes to anabolic resistance in obese sarcopenic old rats through eIF2α activation. Aging Cell. 2014;13(6):1001-1011. doi:10.1111/acel.12263
62. Tezze C, Sandri M, Tessari P. Anabolic Resistance in the Pathogenesis of Sarcopenia in the Elderly: Role of Nutrition and Exercise in Young and Old People. Nutrients. 2023;15(18):4073. Published 2023 Sep 20. doi:10.3390/nu15184073
63. Verstraeten LMG, van Wijngaarden JP, Pacifico J, Reijnierse EM, Meskers CGM, Maier AB. Association between malnutrition and stages of sarcopenia in geriatric rehabilitation inpatients: RESORT. Clin Nutr. 2021;40(6):4090-4096. doi:10.1016/j.clnu.2021.02.007

64. Vieira FT, Godziuk K, Barazzoni R, et al. Hidden malnutrition in obesity and knee osteoarthritis: Assessment, overlap with sarcopenic obesity and health outcomes. Clin Nutr. 2025;48:111-120. doi:10.1016/j.clnu.2025.03.019
65. Wang H, Zhao T, Guo G, et al. Global Leadership Initiative on Malnutrition-defined malnutrition coexisting with visceral adiposity predicted worse long-term all-cause mortality among inpatients with decompensated cirrhosis. Nutr Diabetes. 2024;14(1):76. Published 2024 Sep 27. doi:10.1038/s41387-024-00336-9
66. Wang M, Tan Y, Shi Y, Wang X, Liao Z, Wei P. Diabetes and Sarcopenic Obesity: Pathogenesis, Diagnosis, and Treatments. Front Endocrinol (Lausanne). 2020;11:568. Published 2020 Aug 25. doi:10.3389/fendo.2020.00568
67. Wannamethee SG, Atkins JL. Sarcopenic Obesity and Cardiometabolic Health and Mortality in Older Adults: a Growing Health Concern in an Ageing Population. Curr Diab Rep. 2023;23(11):307-314. doi:10.1007/s11892-023-01522-2
68. Xu Q, Shi RF, Yu XJ, et al. Sarcopenic obesity definitions and their association with cardiovascular disease and mortality. BMC Geriatr. 2026;26(1):709. Published 2026 Apr 10. doi:10.1186/s12877-026-07433-7
69. Yamaguchi M, Ito M, Sugiyama H, et al. Malnutrition according to the GLIM criteria with kidney dysfunction is associated with increased mortality in hospitalized patients with cardiovascular disease: A retrospective cohort study. Clin Nutr ESPEN. 2023;55:167-173. doi:10.1016/j.clnesp.2023.02.029
70. Zoppi CC, Silveira LR, Oliveira CA, Boschero AC, Curi R, Carneiro EM. Insulin release, peripheral insulin resistance and muscle function in protein malnutrition: a role of tricarboxylic acid cycle anaplerosis. Br J Nutr. 2010;103(9):1237-1250. doi:10.1017/S0007114509993060
71. Zuo X, Li X, Tang K, et al. Sarcopenia and cardiovascular diseases: A systematic review and meta-analysis. J Cachexia Sarcopenia Muscle. 2023;14(3):1183-1198. doi:10.1002/jcsm.13221

Supplementary Material

Supplementary tables for study results

Supplementary table R1. Prevalence of sarcopenic obesity among the 237 participants with complete diagnostic data. Data are expressed as numbers (percentage relative to the column totals). P-values were derived using the chi-square test, with statistically significant values highlighted in bold. Abbreviations: ESPEN/EASO, European Society for Clinical Nutrition and Metabolism/European Association for the Study of Obesity. P-values were adjusted for multiple comparisons using the Sidak correction

Classification		Total	Inpatients	Outpatients	p-value
Sarcopenic obesity for 237 participants (ESPEN/EASO)	No sarcopenic obesity	94 (39.7)	56 (32.9)	38 (56.7)	**<0.001**
	Probable sarcopenic obesity	115 (48.5)	86 (50.6)	29 (43.3)	
	Confirmed stage I	0 (0)	0 (0)	0 (0)	
	Confirmed stage II	28 (11.8)	28 (16.5)	0 (0)	

Supplementary table R2. Multivariable logistic regression model of factors associated with sarcopenic obesity considering the 237 participants for whom all the data were available. Odds ratios (ORs), 95% confidence intervals (95% CIs), standard errors, zeta and p-values are reported, with statistically significant p values highlighted in bold. As the centenarian age category comprised only one participant, it could not be reliably included as a level of the age-class variable and was therefore excluded from the model.

	Factors	Odds ratio (95% CI)	Std. err.	z	P-value
Sarcopenic obesity (ESPEN/ EASO)	Age (SIGG age classes)				
	75-84 years	2.08 (1.10–3.90)	0.67	2.26	**0.024**
	85-99 years	0.84 (0.32–2.20)	0.41	-0.35	0.729
	Low physical activity level (PASE < 76)	1.70 (0.88–3.29)	0.57	1.57	0.115
	Setting	0.51 (0.23–1.13)	0.21	-1.65	0.098
	Disease burden (CIRS severity index ≥2.5)	1.19 (0.61–2.32)	0.41	0.52	0.607
	Acute disease	0.92 (0.44–1.93)	0.35	-0.21	0.833

Supplementary table R3. Multivariable logistic regression model of factors associated with sarcopenic diabetes. Odds ratios (ORs), 95% confidence intervals (95% CIs), standard errors, zeta, and p-values are reported, with statistically significant p-values highlighted in bold. As no outpatient subjects with sarcopenic diabetes were included, the setting variable was omitted from the regression model.

	Factors	Odds ratio (95% CI)	Std. err.	z	P-value
Sarcopenic diabetes (sarcopenia and type 2 diabetes)	Age (SIGG age classes)				
	75-84 years	2.89 (0.78–10.72)	1.93	1.59	0.112
	85-99 years	3.68 (0.85–15.87)	2.74	1.75	0.080
	Severity of the disease burden (CIRS severity index ≥ 2.5)	10.55 (2.33–47.75)	8.13	3.06	**0.002**
	Acute disease	2.12 (0.65–6.85)	1.27	1.25	0.210

Supplementary table R4. Multivariable logistic regression model of factors associated with sarcopenic diabetes among inpatients; no outpatient suffered from sarcopenic diabetes. Odds ratios (ORs), 95% confidence intervals (95% CIs), standard errors, zeta, and p-values are reported, with statistically significant p-values highlighted in bold.

	Factors	Odds ratio (95% CI)	Std. err.	z	P-value

Sarcopenic diabetes (sarcopenia and type 2 diabetes)	Age (SIGG age classes)<br>75-84 years<br>85-99 years	 2.77 (0.75–10.23)<br>3.47 (0.81–14.82)	 1.85<br>2.57	 1.53<br>1.68	 0.126<br>0.093
	Severity of the disease burden (CIRS severity index ≥ 2.5)	8.47 (1.92–37.40)	6.42	2.82	**0.003**
	Acute disease	1.70 (0.53–5.45)	1.01	0.90	0.371

Supplementary Table R5. Inflammatory and laboratory biomarkers by sex. Data are expressed as mean (standard deviation); n indicates the number of participants with available data for each biomarker. P-values were derived using parametric tests, except where indicated (*), for which non-parametric tests (Mann–Whitney or Kruskal–Wallis) were used. Abbreviations: CRP, C-reactive protein; IL-6, interleukin-6; IL-10, interleukin-10; TGF-β, transforming growth factor-beta; TNF-α, tumor necrosis factor-alpha; WBC, white blood cell count. P-values were adjusted for multiple comparisons using the Sidak correction.

Blood biomarkers	Men		Women		P-value
	n	Mean (SD)	n	Mean (SD)	
CRP (mg/dL)	90	3.8 (8.8)	108	2.6 (5.5)	0.319
WBC (10^9/L)	150	7.5 (2.4)	150	7.9 (3.2)	0.425
Platelets (10^9/L)	149	204.8 (60.9)	149	245.0 (79.0)	**<0.001**
Prealbumin (mg/dL)	74	22.3 (7.4)	91	21.4 (6.9)	0.453
Uric acid (mg/dL)	67	5.7 (1.6)	84	5.4 (2.2)	0.249
IL-10 (pg/mL)	93	10.7 (37.2)	102	17.3 (96.9)	0.859*
IL-6 (pg/mL)	99	11.9 (23.1)	113	11.7 (18.7)	0.514*
TNF-α (pg/mL)	98	3.0 (10.6)	113	1.3 (5.6)	0.182*
TGF-β (pg/mL)	97	24879.5 (13660.5)	104	26494.4 (15441.7)	0.434

Supplementary table R6. Inflammatory biomarker levels according to presence or absence of metabolic syndrome. Data are presented as mean (standard deviation), and p-values, with statistically significant p-values highlighted in bold; n indicates the number of participants with available data for each biomarker. P-values were derived using parametric tests, except where indicated (*), for which non-parametric tests (Mann–Whitney or Kruskal–Wallis) were used. P-values were adjusted for multiple comparisons using the Sidak correction.

Blood biomarkers	Metabolic Syndrome				p-value
	No		Yes		
	n	mean (SD)	n	mean (SD)	
CRP (mg/dL)	84	1.9 (4.6)	114	4.0 (8.6)	**<0.001**
WBC (10^9/L)	120	7.1 (2.6)	180	8.1 (2.9)	**<0.001**
Platelets (10^9/L)	118	232.9 (67.8)	180	219.6 (76.4)	0.125
Prealbumin (mg/dL)	77	22.6 (6.3)	88	21.1 (7.7)	0.19
Uric acid (mg/dL)	72	5.1 (1.8)	79	6.0 (2.0)	**0.006**
IL-10 (pg/mL)	77	6.9 (19.8)	118	18.9 (94.3)	0.149*
IL-6 (pg/mL)	85	9.8 (19.0)	127	13.2 (21.9)	**0.021***
TNF-α (pg/mL)	84	2.8 (10.7)	127	1.6 (6.3)	0.692*
TGF-β (pg/mL)	78	24929.3 (14391.4)	123	26213.3 (14760.1)	0.544

Supplementary Material

Supplementary table R7. Inflammatory biomarker profile according to presence or absence of sarcopenic obesity. Data are presented as mean (standard deviation) and p-values, with statistically significant p-values highlighted in bold; n indicates the number of participants with available data for each biomarker. P-values were derived using parametric tests, except where indicated (*), for which non-parametric tests (Mann–Whitney or Kruskal–Wallis) were used, or where indicated (†), for which one-tailed t-test (difference in means > 0). P-values were adjusted for multiple comparisons using the Sidak correction.

Blood biomarkers	Sarcopenic obesity (ESPEN/ EASO)				p-value
	No		Yes		
	n	mean (SD)	n	mean (SD)	
CRP (mg/dL)	153	3.1 (7.8)	45	3.1 (5.0)	0.116
WBC (10^9/L)	236	7.7 (2.8)	64	7.7 (2.8)	0.9
Platelets (10^9/L)	234	223.7 (67.4)	64	229.0 (91.9)	0.612
Prealbumin (mg/dL)	127	22.6 (7.0)	38	19.2 (6.9)	**0.01**
Uric acid (mg/dL)	120	5.5 (1.8)	31	5.6 (2.5)	0.736
IL-10 (pg/mL)	154	10.0 (35.9)	41	29.9 (147.3)	0.582*
IL-6 (pg/mL)	167	10.1 (19.8)	45	18.1 (23.3)	**0.002***
TNF-α (pg/mL)	166	2.2 (8.9)	45	1.7 (5.7)	0.769*
TGF-β (pg/mL)	158	26640.0 (14954.3)	43	22316.5 (12783.5)	0.085; **0.0425†**

Supplementary table R8. Inflammatory biomarker concentrations according to adherence to the Mediterranean diet (MEDI-LITE score). Data are presented as mean (standard deviation) and p-values, with statistically significant p-values highlighted in bold; n indicates the number of participants with available data for each biomarker. P-values were derived using parametric tests, except where indicated (*), for which non-parametric tests (Mann–Whitney or Kruskal–Wallis) were used. P-values were adjusted for multiple comparisons using the Sidak correction.

Blood biomarkers	Mediterranean Diet adherence				p-value
	High adherence (Medilite ≥ 8.5)		Low adherence (Medilite < 8.5)		
	n	mean (SD)	n	mean (SD)	
CRP (mg/dL)	184	3.0 (7.2)	12	4.8 (8.1)	0.299
WBC (10^9/L)	279	7.6 (2.6)	19	9.3 (5.0)	**0.024**
Platelets (10^9/L)	277	224.6 (72.2)	19	228.7 (89.7)	0.814
Prealbumin (mg/dL)	156	21.9 (7.1)	9	20.2 (8.3)	0.497
Uric acid (mg/dL)	142	5.5 (2.0)	9	5.7 (1.9)	0.763
IL-10 (pg/mL)	184	14.6 (76.7)	9	6.1 (10.2)	0.989*
IL-6 (pg/mL)	201	11.6 (20.5)	9	17.6 (28.5)	0.875*
TNF-α (pg/mL)	200	2.2 (8.6)	9	0.0 (0.0)	0.378*
TGF-β (pg/mL)	190	25998.0 (14748.3)	9	21351.2 (11061.2)	0.352

Supplementary table R9. Inflammatory biomarker concentrations according to adherence to the MIND diet. Data are presented as mean (standard deviation) and p-values, with statistically significant p-values highlighted in bold; n indicates the number of participants with available data for each biomarker. P-values were derived using parametric tests, except where indicated (*), for which non-parametric tests (Mann–Whitney or Kruskal–Wallis) were used. P-values were adjusted for multiple comparisons using the Sidak correction.

Blood biomarkers	MIND diet adherence (percentile classes)						p-value
	Low adherence		Moderate adherence		High adherence		
	n	mean (SD)	n	mean (SD)	n	mean (SD)	
CRP (mg/dL)	36	3.3 (6.3)	126	3.4 (8.2)	34	2.0 (3.5)	0.471
WBC (10^9/L)	50	8.2 (3.8)	191	7.7 (2.7)	57	7.5 (2.3)	0.599
Platelets (10^9/L)	48	237.5 (61.6)	192	223.2 (77.4)	56	219.7 (67.7)	0.406
Prealbumin (mg/dL)	32	20.3 (7.4)	105	21.9 (7.3)	28	23.0 (5.9)	0.348
Uric acid (mg/dL)	26	6.5 (2.1)	100	5.4 (1.9)	25	5.3 (2.0)	**0.03**
IL-10 (pg/mL)	29	38.3 (174.8)	124	7.7 (25.5)	40	16.9 (55.2)	0.586*
IL-6 (pg/mL)	29	10.7 (14.2)	136	11.9 (21.4)	45	12.4 (23.0)	0.777*
TNF-α (pg/mL)	29	0.5 (2.5)	135	2.3 (8.2)	45	2.5 (11.1)	0.562*
TGF-β (pg/mL)	27	25402.1 (12429.3)	129	25975.0 (15029.2)	43	25468.4 (14906.3)	0.970

Supplementary table R10. Inflammatory biomarker concentrations according to wine, beer, and aperitif consumption habits. Data are presented as mean (standard deviation) and p-values, with statistically significant p-values highlighted in bold; n indicates the number of participants with available data for each biomarker. P-values were derived using parametric tests, except where indicated (*), for which non-parametric tests (Mann–Whitney or Kruskal–Wallis) were used. P-values were adjusted for multiple comparisons using the Sidak correction.

Blood biomarkers	Alcohol consumption: wine, beer, aperitifs						p-value
	never		occasional (< once per week)		habitual (≥ once per week)		
	n	mean (SD)	n	mean (SD)	n	mean (SD)	
CRP (mg/dL)	9	11.7 (21.6)	45	2.3 (6.0)	86	2.8 (5.8)	0.424
WBC (10^9/L)	13	6.8 (1.6)	72	7.3 (2.2)	132	7.6 (2.6)	0.469
Platelets (10^9/L)	13	183.1 (77.4)	74	230.8 (61.7)	129	218.7 (65.6)	**0.046**
Prealbumin (mg/dL)	7	22.4 (9.0)	44	20.5 (5.8)	72	23.2 (8.2)	0.179
Uric acid (mg/dL)	7	4.2 (1.2)	35	5.4 (1.9)	71	5.6 (1.8)	0.153
IL-10 (pg/mL)	7	4.8 (6.7)	45	6.7 (13.3)	89	12.6 (46.3)	0.234*
IL-6 (pg/mL)	9	4.5 (4.0)	52	16.7 (28.7)	94	9.7 (16.2)	0.22
TNF-α (pg/mL)	9	4.1 (8.1)	52	1.1 (5.5)	93	2.7 (10.7)	0.167
TGF-β (pg/mL)	9	23523.8 (9870.1)	48	26869.8 (15518.2)	91	26323.6 (14155.6)	0.815

Supplementary table R11. Inflammatory biomarker concentrations according to spirits consumption habits. Data are presented as mean (standard deviation) and p-values, with statistically significant p-values highlighted in bold; n indicates the number of participants with available data for each biomarker. P-values were derived using parametric tests, except where indicated (*), for which non-parametric tests (Mann–Whitney or Kruskal–Wallis) were used. P-values were adjusted for multiple comparisons using the Sidak correction.

Blood biomarkers	Alcohol consumption: spirits						p-value
	never		occasional (< once per week)		habitual (≥ once per week)		
	n	mean (SD)	n	mean (SD)	n	mean (SD)	
CRP (mg/dL)	65	3.7 (9.8)	60	2.9 (6.5)	14	2.2 (3.5)	0.81

Supplementary Material

WBC (10^9/L)	86	7.5 (2.8)	105	7.4 (2.3)	25	7.3 (1.7)	0.988
Platelets (10^9/L)	88	212.5 (64.7)	103	228.1 (69.0)	24	222.3 (51.7)	0.261
Prealbumin (mg/dL)	49	21.5 (6.6)	59	22.2 (8.1)	13	24.2 (8.4)	0.538
Uric acid (mg/dL)	47	5.4 (2.0)	54	5.5 (1.8)	10	5.9 (1.6)	0.754
IL-10 (pg/mL)	59	4.9 (8.5)	65	17.1 (54.0)	16	3.7 (8.6)	0.474*
IL-6 (pg/mL)	66	14.9 (25.4)	71	8.7 (14.6)	17	12.3 (25.4)	0.241*
TNF-α (pg/mL)	66	1.5 (6.0)	70	2.1 (9.3)	17	5.7 (16.1)	0.670*
TGF-β (pg/mL)	61	24949.6 (13730.8)	69	27666.8 (15415.0)	17	26036.2 (12491.0)	0.561
