# Supplementary material for: Prevalence and clinical characterization of malnutrition, sarcopenia and sarcopenic obesity in older adults with cardiometabolic diseases: SENIOR cross-sectional study
Source: Front Nutr. 2026 Oct 28;13:1927628. doi: 10.3389/fnut.2026.1927628 (PMC13753960; doi:10.3389/fnut.2026.1927628)
Supplement: Supplementary file 2 [file Table_1.pdf]

Supplementary Material

SENIOR (Sustainable Eating patterN to lImit malnutritiOn in oldeR adults) – cross-sectional

1. Table S1. STROBE-nut checklist

STROBE-nut 2016: An extension of the STROBE statement for nutritional epidemiology
Reference: Lachat C et al. (2016). Strengthening the Reporting of Observational Studies in Epidemiology-Nutritional Epidemiology (STROBE-nut): An Extension of the STROBE Statement. PLoS Med;13:e1002036.

Item	Item nr	STROBE recommendations	Extension for Nutritional Epidemiology studies (STROBE-nut)	Reported on page #
**Title and abstract**	1	(a) Indicate the study's design with a commonly used term in the title or the abstract.<br>(b) Provide in the abstract an informative and balanced summary of what was done and what was found.	**nut-1** State the dietary/nutritional assessment method(s) used in the title, abstract, or keywords.	**1-2**
**Introduction**				
Background rationale	2	Explain the scientific background and rationale for the investigation being reported.		**2-3**
Objectives	3	State specific objectives, including any pre-specified hypotheses.		**2-3**
**Methods**				
Study design	4	Present key elements of study design early in the paper.		**3**
Settings	5	Describe the setting, locations, and relevant dates, including periods of recruitment, exposure, follow-up, and data collection.	**nut-5** Describe any characteristics of the study settings that might affect the dietary intake or nutritional status of the participants, if applicable.	**3**

Item	Item nr	STROBE recommendations	Extension for Nutritional Epidemiology studies (STROBE-nut)	Reported on page #
Participants	6	a) Cohort study—Give the eligibility criteria, and the sources and methods of selection of participants. Describe methods of follow-up.<br>Case-control study—Give the eligibility criteria, and the sources and methods of case ascertainment and control selection. Give the rationale for the choice of cases and controls.<br>Cross-sectional study—Give the eligibility criteria, and the sources and methods of selection of participants.<br>(b) Cohort study—For matched studies, give matching criteria and number of exposed and unexposed.<br>Case-control study—For matched studies, give matching criteria and the number of controls per case.	**nut-6** Report particular dietary, physiological or nutritional characteristics that were considered when selecting the target population.	**3-4**
Variables	7	Clearly define all outcomes, exposures, predictors, potential confounders, and effect modifiers. Give diagnostic criteria, if applicable.	**nut-7.1** Clearly define foods, food groups, nutrients, or other food components.<br>**nut-7.2** When using dietary patterns or indices, describe the methods to obtain them and their nutritional properties.	**4-5 and Suppleme ntary materials**
Data sources - measurements	8	For each variable of interest, give sources of data and details of methods of assessment (measurement).Describe comparability of assessment methods if there is more than one group.	**nut-8.1** Describe the dietary assessment method(s), e.g., portion size estimation, number of days and items recorded, how it was developed and administered, and how quality was assured. Report if and how supplement intake was assessed.<br>**nut-8.2** Describe and justify food composition	**4-5 and Suppleme ntary materials; please see Table S2 for the STROBE-ME checklist**

Item	Item nr	STROBE recommendations	Extension for Nutritional Epidemiology studies (STROBE-nut)	Reported on page #
			data used. Explain the procedure to match food composition with consumption data. Describe the use of conversion factors, if applicable. **nut-8.3** Describe the nutrient requirements, recommendations, or dietary guidelines and the evaluation approach used to compare intake with the dietary reference values, if applicable. **nut-8.4** When using nutritional biomarkers, additionally use the STROBE Extension for Molecular Epidemiology (STROBE-ME). Report the type of biomarkers used and their usefulness as dietary exposure markers. **nut-8.5** Describe the assessment of nondietary data (e.g., nutritional status and influencing factors) and timing of the assessment of these variables in relation to dietary assessment. **nut-8.6** Report on the validity of the dietary or nutritional assessment methods and any internal or external validation used in the study, if applicable.	
Bias	9	Describe any efforts to address potential sources of bias.	**nut-9** Report how bias in dietary or nutritional assessment was addressed, e.g., misreporting, changes in habits as a result of being measured, or data	**3-5; 13**

**Item**	**Item nr**	**STROBE recommendations**	**Extension for Nutritional Epidemiology studies (STROBE-nut)**	**Reported on page #**
			imputation from other sources	
Study Size	10	Explain how the study size was arrived at.		**5**
Quantitative variables	11	Explain how quantitative variables were handled in the analyses. If applicable, describe which groupings were chosen and why.	**nut-11** Explain categorization of dietary/nutritional data (e.g., use of N-tiles and handling of nonconsumers) and the choice of reference category, if applicable.	**5 and Suppleme ntary materials**
Statistical Methods	12	(a) Describe all statistical methods, including those used to control for confounding<br>(b) Describe any methods used to examine subgroups and interactions.<br>(c) Explain how missing data were addressed.<br>(d) Cohort study—If applicable, explain how loss to follow-up was addressed.<br>Case-control study—If applicable, explain how matching of cases and controls was addressed.<br>Cross-sectional study—If applicable, describe analytical methods taking account of sampling strategy.<br>(e) Describe any sensitivity analyses.	**nut-12.1** Describe any statistical method used to combine dietary or nutritional data, if applicable.<br>**nut-12.2** Describe and justify the method for energy adjustments, intake modeling, and use of weighting factors, if applicable.<br>**nut-12.3** Report any adjustments for measurement error, i.e,. from a validity or calibration study.	**5**
**Results**				
Participants	13	(a) Report the numbers of individuals at each stage of the study—e.g., numbers potentially eligible, examined for eligibility, confirmed eligible, included in the study, completing follow-up, and analyzed.<br>(b) Give reasons for non-participation at each stage.	**nut-13** Report the number of individuals excluded based on missing, incomplete or implausible dietary/nutritional data.	**5 and flow diagram in the Suppleme ntary materials**

Item	Item nr	STROBE recommendations	Extension for Nutritional Epidemiology studies (STROBE-nut)	Reported on page #
		(c) Consider use of a flow diagram.		
Descriptive data	14	(a) Give characteristics of study participants (e.g., demographic, clinical, social) and information on exposures and potential confounders<br>(b) Indicate the number of participants with missing data for each variable of interest<br>(c) Cohort study—Summarize follow-up time (e.g., average and total amount)	**nut-14** Give the distribution of participant characteristics across the exposure variables if applicable. Specify if food consumption of total population or consumers only were used to obtain results.	**5-7 and Suppleme ntary materials for missing data**
Outcome data	15	Cohort study—Report numbers of outcome events or summary measures over time.<br>Case-control study—Report numbers in each exposure category, or summary measures of exposure.<br>Cross-sectional study—Report numbers of outcome events or summary measures.		**8**
Main results	16	(a) Give unadjusted estimates and, if applicable, confounder-adjusted estimates and their precision (e.g., 95% confidence interval).<br>Make clear which confounders were adjusted for and why they were included.<br>(b) Report category boundaries when continuous variables were categorized.<br>(c) If relevant, consider translating estimates of relative risk into absolute risk for a meaningful time period.	**nut-16** Specify if nutrient intakes are reported with or without inclusion of dietary supplement intake, if applicable.	**8-12**
Other analyses	17	Report other analyses done—e.g., analyses of subgroups and interactions and sensitivity analyses.	**nut-17** Report any sensitivity analysis (e.g., exclusion of misreporters or outliers) and data imputation, if applicable.	/
**Discussion**				

Item	Item nr	STROBE recommendations	Extension for Nutritional Epidemiology studies (STROBE-nut)	Reported on page #
Key results	18	Summarize key results with reference to study objectives.		**12-13**
Limitation	19	Discuss limitations of the study, taking into account sources of potential bias or imprecision. Discuss both direction and magnitude of any potential bias.	**Nut-19** Describe the main limitations of the data sources and assessment methods used and implications for the interpretation of the findings.	**13**
Interpretation	20	Give a cautious overall interpretation of results considering objectives, limitations, multiplicity of analyses, results from similar studies, and other relevant evidence.	**nut-20** Report the nutritional relevance of the findings, given the complexity of diet or nutrition as an exposure.	**12-13**
Generalizability	21	Discuss the generalizability (external validity) of the study results.		**12-13**
**Other information**				
Funding	22	Give the source of funding and the role of the funders for the present study and, if applicable, for the original study on which the present article is based.		**14**
Ethics			**nut-22.1** Describe the procedure for consent and study approval from ethics committee(s).	**14**
Supplementary material			**nut-22.2** Provide data collection tools and data as online material or explain how they can be accessed.	**15**

2. Table S2. STROBE-ME checklist

STROBE-ME 2016: An extension of the STROBE statement for molecular epidemiology
Reference: Gallo V, Egger M, McCormack V, et al. STrengthening the Reporting of OBservational studies in Epidemiology - Molecular Epidemiology (STROBE-ME): an extension of the STROBE statement. Eur J Clin Invest. 2012;42:1-16.

**Item**	**Item nr**	**STROBE recommendations**	**Extension for Molecular EpidemiologyStudies (STROBE-ME)**	**Reported on page #**
**Title and abstract**	1	(a) Indicate the study's design with a commonly used term in the title or the abstract.<br>(b) Provide in the abstract an informative and balanced summary of what was done and what was found.	**ME-1** State the use of specific biomarker(s) in the title and/or in the abstract if they contribute substantially to the findings	**1-2**
**Introduction**				
Background rationale	2	Explain the scientific background and rationale for the investigation being reported.	**ME-2** Explain in the scientific background of the paper how/why the specific biomarker(s) have been chosen,potentially among many others (e.g. others are studied but reported elsewhere or not studied at all)	**2-3 and Suppleme ntary materials**
Objectives	3	State specific objectives, including any pre-specified hypotheses.	**ME-3** A priori hypothesis: if one or more biomarkers are used as proxy measures, state the a priori hypothesis on the expected values of the biomarker(s)	/
**Methods**				
Study design	4	Present key elements of study design early in the paper.	**ME-4** Describe the special study designs for molecular epidemiology (in particular, nested case/control and case/cohort) and how they were implemented	**3-4 and Suppleme ntary materials**
Biological sample collection			**ME-4.1** Report on the setting of the biological sample collection; amount of sample; nature of collecting procedures; participant conditions; time between sample collection	

Item	Item nr	STROBE recommendations	Extension for Molecular EpidemiologyStudies (STROBE-ME)	Reported on page #
			and relevant clinical or physiological endpoints	
Biological sample storage			**ME-4.2** Describe sample processing (centrifugation, timing, additives, etc.)	
Biological sample processing			**ME-4.3** Describe sample storage until biomarker analysis (storage, thawing, manipulation, etc.)	
Biomarker biochemical characteristics			**ME-4.4** Report the half-life of the biomarker and chemical and physical characteristics (e.g. solubility)	
Settings	5	Describe the setting, locations, and relevant dates, including periods of recruitment, exposure, follow-up, and data collection.		
Participants	6	a) Cohort study—Give the eligibility criteria, and the sources and methods of selection of participants. Describe methods of follow-up.<br>Case-control study—Give the eligibility criteria, and the sources and methods of case ascertainment and control selection. Give the rationale for the choice of cases and controls.<br>Cross-sectional study—Give the eligibility criteria, and the sources and methods of selection of participants.<br>(b) Cohort study—For matched studies, give matching criteria and number of exposed and unexposed.<br>Case-control study—For matched studies, give matching criteria and the number of controls per case.	**ME-6** Report any habit, clinical condition, physiological factor or working or living condition that might affect the characteristics or concentrations of the biomarker	/
Variables	7	Clearly define all outcomes, exposures, predictors, potential		

Item	Item nr	STROBE recommendations	Extension for Molecular EpidemiologyStudies (STROBE-ME)	Reported on page #
		confounders, and effect modifiers. Give diagnostic criteria, if applicable.		
Data sources - measurements	8	For each variable of interest, give sources of data and details of methods of assessment (measurement).Describe comparability of assessment methods if there is more than one group.	**ME-8** Laboratory methods: report type of assay used, detection limit, quantity of biological sample used, outliers, timing in the assay procedures (when applicable) and calibration procedures or any standard used	**Suppleme ntary materials**
Bias	9	Describe any efforts to address potential sources of bias.		
Study Size	10	Explain how the study size was arrived at.		
Quantitative variables	11	Explain how quantitative variables were handled in the analyses. If applicable, describe which groupings were chosen and why.		
Statistical Methods	12	(a) Describe all statistical methods, including those used to control for confounding<br>(b) Describe any methods used to examine subgroups and interactions.<br>(c) Explain how missing data were addressed.<br>(d) Cohort study—If applicable, explain how loss to follow-up was addressed.<br>Case-control study—If applicable, explain how matching of cases and controls was addressed.<br>Cross-sectional study—If applicable, describe analytical methods taking account of sampling strategy.<br>(e) Describe any sensitivity analyses.	**ME-12** Describe how biomarkers were introduced into statistical models	**5**
Validity/reliability of measurement and			**ME-12.1** Report on the validity and reliability of	**2-3**

Item	Item nr	STROBE recommendations	Extension for Molecular EpidemiologyStudies (STROBE-ME)	Reported on page #
internal/external validation			measurement of the biomarker(s) coming from the literature and any internal or external validation used in the study	
**Results**				
Participants	13	(a) Report the numbers of individuals at each stage of the study—e.g., numbers potentially eligible, examined for eligibility, confirmed eligible, included in the study, completing follow-up, and analyzed.<br>(b) Give reasons for non-participation at each stage.<br>(c) Consider use of a flow diagram.	**ME-13** Give reason for loss of biological samples at each stage	**5 and Suppleme ntary materials for missing data**
Descriptive data	14	(a) Give characteristics of study participants (e.g., demographic, clinical, social) and information on exposures and potential confounders<br>(b) Indicate the number of participants with missing data for each variable of interest<br>(c) Cohort study—Summarize follow-up time (e.g., average and total amount)	**ME-14.1** Give the distribution of the biomarker measurement (including mean, median, range and variance)	**10-12**
Outcome data	15	Cohort study—Report numbers of outcome events or summary measures over time.<br>Case-control study—Report numbers in each exposure category, or summary measures of exposure.<br>Cross-sectional study—Report numbers of outcome events or summary measures.		
Main results	16	(a) Give unadjusted estimates and, if applicable, confounder-adjusted estimates and their precision (e.g., 95% confidence interval).		

Item	Item nr	STROBE recommendations	Extension for Molecular EpidemiologyStudies (STROBE-ME)	Reported on page #
		Make clear which confounders were adjusted for and why they were included.<br>(b) Report category boundaries when continuous variables were categorized.<br>(c) If relevant, consider translating estimates of relative risk into absolute risk for a meaningful time period.		
Other analyses	17	Report other analyses done—e.g., analyses of subgroups and interactions and sensitivity analyses.		
**Discussion**				
Key results	18	Summarize key results with reference to study objectives.		
Limitation	19	Discuss limitations of the study, taking into account sources of potential bias or imprecision. Discuss both direction and magnitude of any potential bias.	**ME-19** Describe main limitations in laboratory procedures	**13**
Interpretation	20	Give a cautious overall interpretation of results considering objectives, limitations, multiplicity of analyses, results from similar studies, and other relevant evidence.	**ME-20** Give an interpretation of results in terms of a priori biological plausibility	**10-12**
Generalizability	21	Discuss the generalizability (external validity) of the study results.		
**Other information**				
Funding	22	Give the source of funding and the role of the funders for the present study and, if applicable, for the original study on which the present article is based.		
Ethics			**ME-22.1** Describe informed consent and approval from ethical committee(s). Specify whether samples were	**3,14**

Item	Item nr	STROBE recommendations	Extension for Molecular EpidemiologyStudies (STROBE-ME)	Reported on page #
			anonymous, anonymized or identifiable	
Supplementary material				
